# Crystallization from solution versus mechanochemistry to obtain double-drug multicomponent crystals of ethacridine with salicylic/acetylsalicylic acids

**DOI:** 10.1038/s41598-023-49922-4

**Published:** 2024-01-21

**Authors:** Artur Mirocki, Mattia Lopresti, Luca Palin, Eleonora Conterosito, Emilia Sikorska, Artur Sikorski, Marco Milanesio

**Affiliations:** 1 Faculty of Chemistry, University of Gdansk, ul. Wita Stwosza 63, 80-308 Gdansk, Poland; 2 Dipartimento di Scienze e Innovazione Tecnologica, Università del Piemonte Orientale, Viale T. Michel 11, 15121 Alessandria, Italy; 3 Nova Res s.r.l., Via D. Bello 3, 28100 Novara, Italy; 4 Dipartimento per lo Sviluppo Sostenibile e la Transizione Ecologica, Università del Piemonte Orientale, Piazza Sant’Eusebio 5, 13100 Vercelli, Italy

**Keywords:** Green chemistry, Materials chemistry

## Abstract

Salicylic and acetylsalicylic acids and ethacridine have complementary bioactive properties. They can be combined to obtain double-drug multicomponent crystals. Their reactivity in different environments was explored to obtain the possible compounds, stable at different hydration degrees. Solution, liquid-assisted grinding, and dry preparation approaches were applied to the couples of reactants in different stoichiometric ratios. Four compounds were obtained, and three out of them were stable and reproducible enough to determine their structures using SCXRD or PXRD methods. When coupled to ethacridine, salicylic acid gave two stable structures (**1** and **3**, both showing 1:1 ratio but different hydration degree) and a metastable one (**5**), while acetylsalicylic acid only one structure from solution (**2** in 1:1 ratio), while LAG caused hydrolysis and formation of the same compound obtained by LAG of ethacridine with salicylic acid. While solution precipitation gave dihydrated (**1**) or monohydrated (**2**) structures with low yields, LAG of salicylic acid and ethacridine allowed obtaining an anhydrous salt complex (**3**) with a yield close to 1. The structures obtained by solution crystallizations maximize π_(acridine)_–π_(acridine)_ contacts with a less compact packing, while the LAG structure is more compact with a packing driven by hydrogen bonds. For all compounds, NMR, ATR-FTIR, and Hirshfeld surface analysis and energy framework calculations were performed.

## Introduction

Acetylsalicylic acid (2-acetoxybenzoic acid, aspirin) represents Nonsteroidal Anti-Inflammatory Drug (NSAID) with an analgesic, antipyretic and anticoagulant activities. Acetylsalicylic acid selectively inhibits of cyclooxygenase enzymes (COXs), as well as prostaglandin synthesis^1–4^. This is a prodrug which hydrolyses into Active Pharmaceutical Ingredient (API) i.e. salicylic acid.

From the crystal engineering and pharmacy points of view, salicylic acid derivatives, as well as other benzoic acids are a good model coformers with pharmaceutical importance used for cocrystal/salt screening which has been extensively described in the literature^5,6^. For example: Berry et al.^7^ investigated cocrystal screening of nicotinamide with seven active pharmaceutical ingredients (ibuprofen, fenbufen, flurbiprofen, ketoprofen, paracetamol, piracetam, and salicylic acid). Manin et al.^8^ reported cocrystal screening of hydroxybenzamides with benzoic acid derivatives, such as 2-,3- and 4-hydroxybenzamide and benzoic, salicylic, acetylsalicylic, 2-,3- and 4-acetamidobenzoic acids . Žegarac et al.^9^ described cocrystal salt formed from sildenafil with salicylic and acetylsalicylic acids, which exhibits an enhanced intrinsic dissolution rate. Lee et al.^10^ reported the formation of cocrystals of salicylic acid with N‐containing bases: 4,4′dipyridyl, nicotinamide, isonicotinamide, N,N′-diacetylpiperazine and piperazine. Zhou et al.^11^ described cocrystals of salicylic acid with benzamide and isonicotinamide with stoichiometric diversity of salicylic acid. Przybyłek et al.^12^ reported on the screening of urea cocrystallization with aromatic carboxylic acids—benzoic acid, salicylic acid, acetylsalicylic acid, 3- and 4-hydroxybenzoic acids, and dihydroxybenzoic acids. Veith et al.^13^ used a thermodynamic approach for co-crystal screening on example cocrystallization carbamazepine with salicylic acid and acetylsalicylic acid. Carneiro et al.^14^ described synthesis and structural characterization of a drug–drug cocrystal flucytosine-acetylsalicylic acid.

In our earlier works, we show that benzoic acid derivatives are also good conformer for screening cocrystal/salt/solvate formation of acridines with pharmaceutical importance, such as acridine^15^, 9-aminoacridine^16^, acriflavine^17^, or 6,9-diamino-2-ethoxyacridine (ethacridine)^18–20^. This group of APIs have an interesting biological activity, such as anticancer, antibacterial, and antiviral and other^21–23^. Especially, ethacridine is commercially available bacteriostatic antiseptic drug, used in the treatment of suppurating infections and infections of the mouth and throat^24,25^.

Multicomponent crystals (cocrystals, salts, salt cocrystals and their solvates), involving one or more API, gained increasing interest from the pharmaceutical industry. In this way, the physicochemical properties of API can be modified by properly selecting coformer or solvent molecules, which are important during drug formulation^26,27^. Such a strategy may also lead to drug synergism^28^. The preparation method plays a key role in the design of multicomponent crystals containing API. Different methods can be used to synthesize such crystals, such as crystallization from solution, liquid-assisted grinding (LAG), dry preparation, or solid-state thermal approach^29^. Depending on the synthesis method and the type and amount of solvent/solvent mixture used for the reaction, it is possible to obtain crystals with different stoichiometric ratios of the components or different polymorphic forms^30–34^.

**Figure 1:**
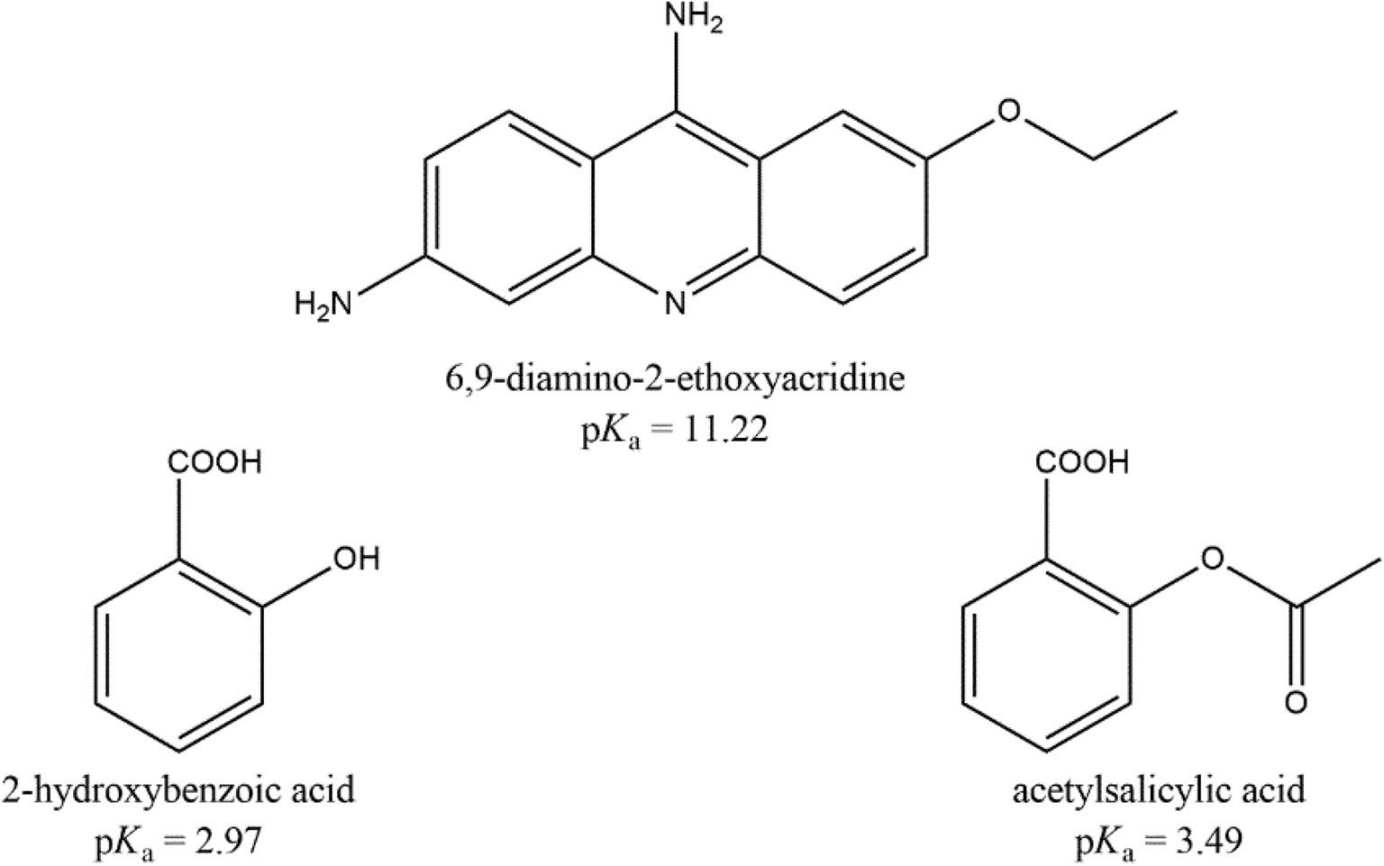
Molecular structures of ethacridine and salicylic acids reported in the article.

In this article, the results of research for the double-drug salts formed from ethacridine and salicylic/acetylsalicylic acids (Fig. 1), drugs with complementary antiseptic properties are described. Reactivity of these APIs in different environments was explored to obtain the possible compounds, stable at different hydration degrees, exploiting previously^19^ developed protocols. The role of water in driving the crystallization was explored applying the solution, LAG and dry grinding approaches to the couples of reactants (Fig. 1) in different stoichiometric ratios. To determine the crystal structures of the obtained compounds, Single-Crystal X-Ray Diffraction (SCXRD) and Powder X-Ray Diffraction (PXRD) measurements were performed. All compounds were characterized using NMR and ATR-FTIR, moreover the Hirshfeld surface analysis and energy framework calculations were carried out. The calculations of the Hirshfeld surfaces, fingerprint plots and energy framework have then been used in a predictive way, to understand the position of a hydrogen atom, as done in our previous work^20^. This was done to assess, with a tool independent from powder diffraction, the ionic or molecular nature of the compound.

## Experimental

### Synthesis and crystallization from solution and LAG

All the chemical compounds were purchased from Sigma-Aldrich and used without further purification.

a) 6,9-Diamino-2-ethoxyacridinium 2-hydroxybenzoate dihydrate (compound **1**).

6,9-Diamino-2-ethoxyacridine-DL-lactate monohydrate (0.05 g, 0.138 mmol) and.

**Figure 2:**
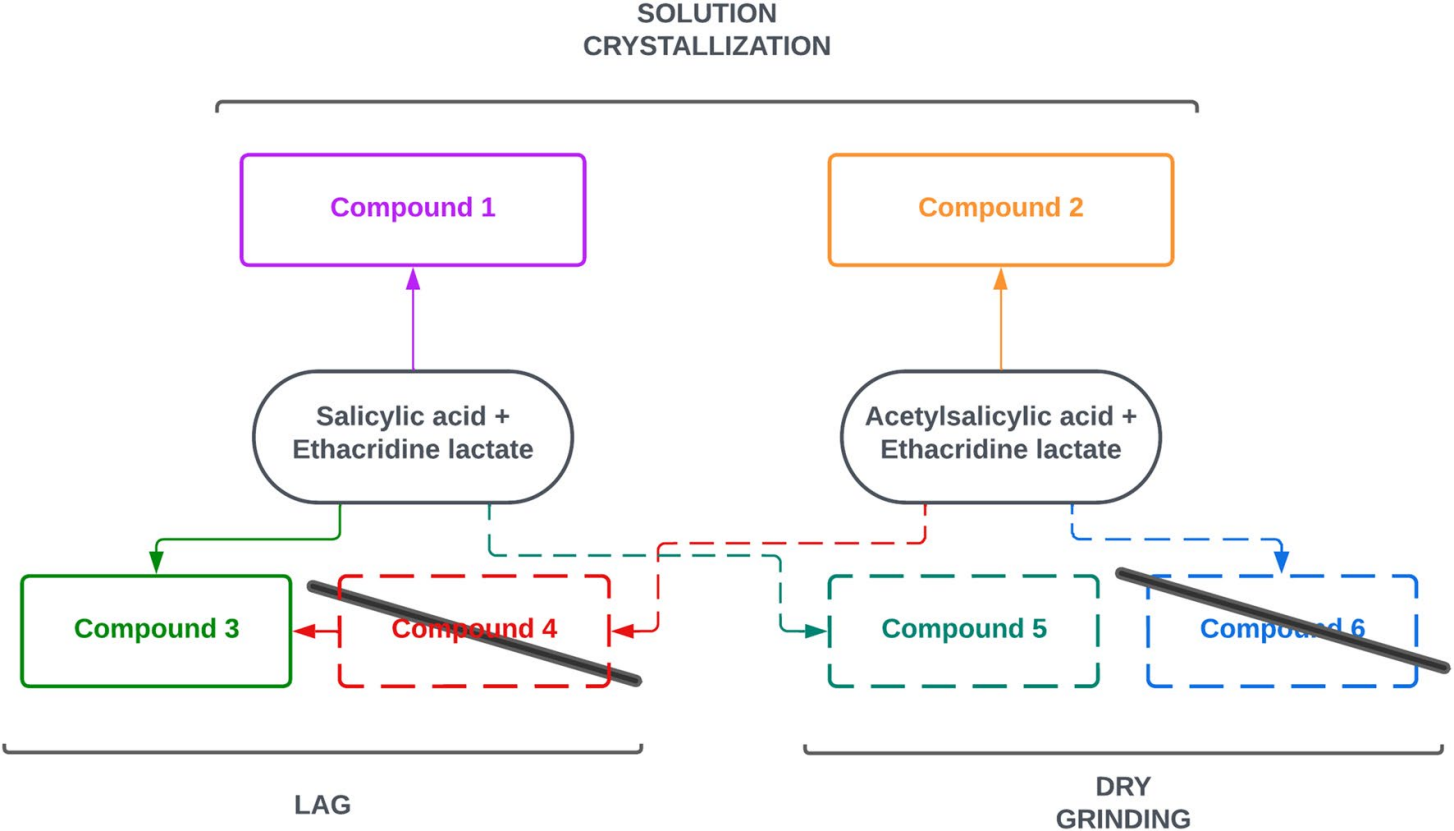
The landscape of obtained (continuous line) or not (dashed lines) crystal structures by the different preparation methods.

**Figure 3:**
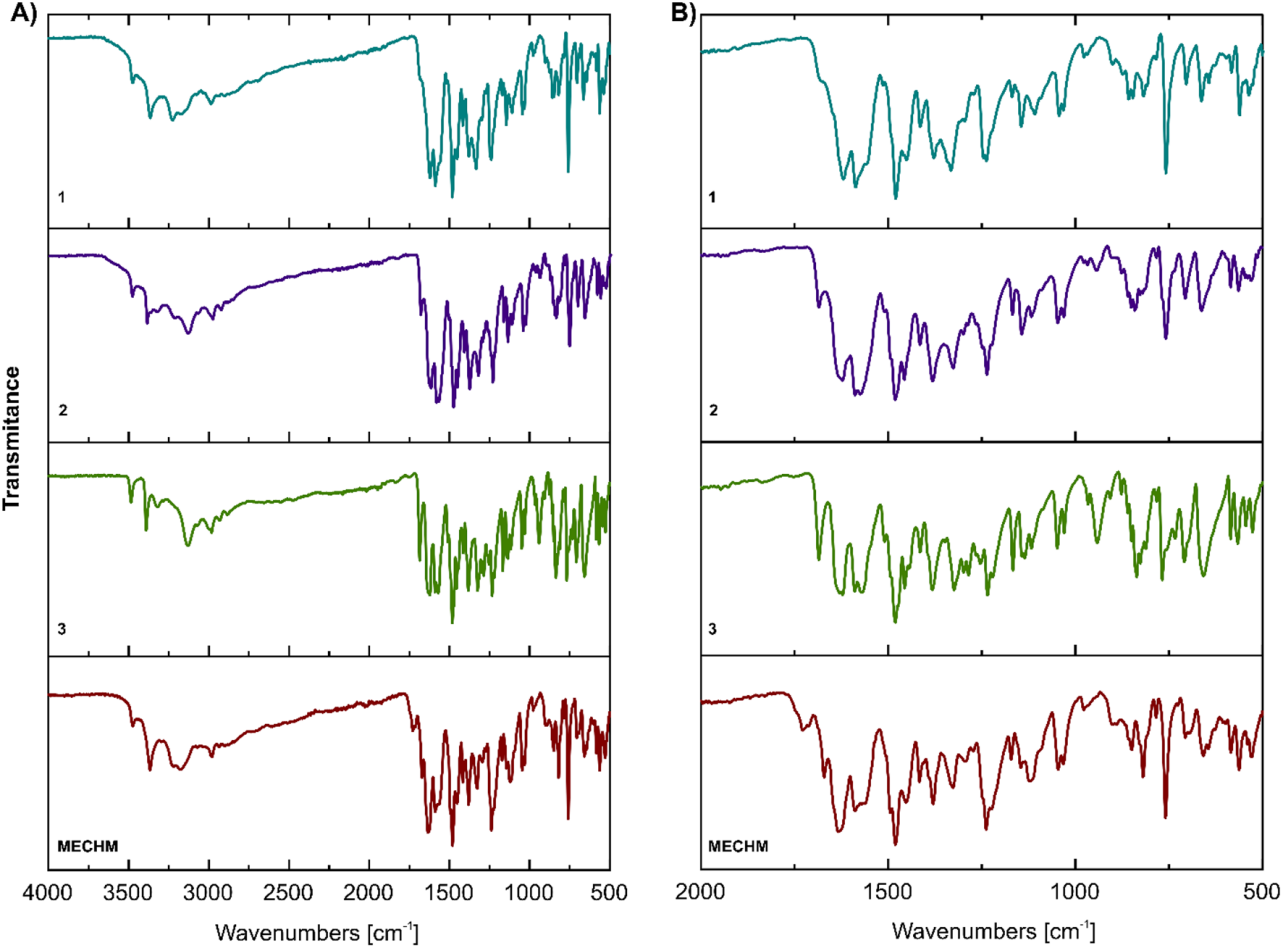
ATR-FTIR spectra of studied compounds in the range of 4000–500 cm^−1^ (**A**) with spectra enlargement in the range of 2000–500 cm^−1^ (**B**).

2-hydroxybenzoic acid (0.011 g, 0.08 mmol) were dissolved in 3 mL of an ethanol/water mixture (2:1 v/v) and heated for 15 min to dissolve the sample. The solution was allowed to evaporate for a few days to give yellow crystals (**1**). ^1^H NMR (500 MHz, DMSO-d6) δ 12.80 (bs, 1H, COOH or OH), 8.98 (s, 2H, NH_2_), 8.24 (d, *J* = 9.2 Hz, 1H, H4), 7.87 (d, *J* = 2.5 Hz, 1H, H5), 7.66 (m, 2H, H8 and H25), 7.53 (dd, *J* = 9.2, 2.5 Hz, 1H, H7), 7.11 (td, *J* = 7.4, 1.8 Hz, 1H, H23), 6.88 (d, *J* = 9.2, 1H, H3), 6.84 (s, 2H, NH_2_), 6.63 (s, 1H, H1), 6.60 (d, *J* = 8.2 Hz, 1H, H22), 6.57 (t, *J* = 7.4 Hz, 1H, H24), 4.17 (q, *J* = 6.9 Hz, 2H, CH_2_), 1.41 (t, *J* = 6.9 Hz, 3H, CH_3_) (Fig. 1S). ^13^C NMR (126 MHz, DMSO-d6) δ 171.80 (C=O), 163.53 (C21), 155.23 (C2), 154.52 (C9), 142.38 (C6), 134.89 (C12 or C14), 131.69 (C12 or C14), 131.64 (C23), 130.35 (C25), 126.37 (C4), 126.30 (C7), 121.13 (C20), 120.16 (C8), 116.42 (C3), 116.22 (C24), 116.16 (C22), 112.35 (C13), 104.65 (C5), 103.38 (C11), 94.76 (C1), 64.45 (CH_2_), 15.00 (CH_3_) (Fig. 2S). ATR-FTIR: 3475 and 3364 cm^−1^ (ν_as and_ ν_sym_ NH_2_), 3290 and 2000 cm^−1^ (νOH, νNH^+^/overtone and combination bands, νCH), 1678 cm^−1^ (shoulder band, ν_as_C=O salicylic acid), 1621–1453 cm^−1^ (νC=C, νC=N, NH_2_ and C-H in-plane bend), 1381 cm^−1^ (ν_sym_C=O, salicylic acid), 1333 (OH in-plane bend), 1238–1031 cm^−1^ (νC–N and νC–O), 759–706 cm^−1^ (=C–H out-of-plane bend), 664 cm^−1^ (in-plane ring deformation) (Fig. 3).

b) 6,9-Diamino-2-ethoxyacridinium 2-acetoxybenzoate monohydrate (compound **2**).

6,9-Diamino-2-ethoxyacridine-DL-lactate monohydrate (0.03 g, 0.083 mmol) and 2-acetoxybenzoic acid (0.015 g, 0.083 mmol) were dissolved in 3 mL of an ethanol/water mixture (2:1 v/v) and heated for 15 min to dissolve the sample. The solution was allowed to evaporate for a few days to give yellow crystals (**2**). ^1^H NMR (500 MHz, DMSO-d6) δ 13.11 (bs, 1H, COOH or OH), 9.00 (s, 2H, NH_2_), 8.25 (d, *J* = 9.2 Hz, 1H, H4), 7.87 (d, *J* = 2.5 Hz, 1H, H5), 7.70 (d, *J* = 9.2 Hz, 1H, H8), 7.66 (dd, *J* = 7.6, 1.8 Hz, 1H, H25), 7.52 (dd, *J* = 9.2, 2.5 Hz, 1H, H7), 7.11 (td, *J* = 7.6, 1.8 Hz, 1H, H23), 6.87 (dd, *J* = 9.2, 2.1 Hz, 1H, H3), 6.81 (s, 2H, NH_2_), 6.64 (d, *J* = 2.1 Hz, 1H, H1), 6.60 (d, *J* = 8.2 Hz, 1H, H22), 6.57 (t, *J* = 7.6 Hz, 1H, H24), 4.16 (q, *J* = 6.9 Hz, 2H, CH_2_), 2.20 (s, 3H, CH_3_), 1.40 (t, *J* = 7.0 Hz, 3H) (Fig. 1S). ^13^C NMR (126 MHz, DMSO-d6) δ 171.73 (C=O), 169.71 (C=O), 163.56 (C21), 155.14 (C2), 154.49 (C9), 142.44 (C6), 135.06 (C12 or C14), 131.70 (C12 or C14), 131.62 (C23), 130.34 (C25), 126.29 (C4), 125.80 (C7), 121.17 (C20), 120.28 (C8), 116.40 (C3), 116.22 (C24), 116.13 (C22), 112.34 (C13), 104.60 (C5), 103.42 (C11), 94.86 (C1), 64.43 (CH_2_), 21.60 (CH_3_–C=O), 15.00 (CH_3_) (Fig. 2S). ATR-FTIR: 3474 and 3368 cm^−1^ (ν_as and_ ν_sym_ NH_2_), 3356–2000 cm^−1^ (νOH, νNH^+^/overtone and combination bands, νCH), 1671 cm^−1^ (ν_as_C=O, salicylic acid), 1634–1456 cm^−1^ (ν_C=C_ and ν_C=N,_ NH_2_ and C–H in-plane bend), 1381 cm^−1^ (ν_sym_C=O, acetylosalicylic acid), 1326 (OH in-plane bend), 1236–1031 cm^−1^ (νC-N and νC-O), 942 cm^−1^ (OH out-of plane bend), 759–706 cm^−1^ (=C–H out-of-plane bend), 663 cm^−1^ (in-plane ring deformation) (Fig. 3).

c) 6,9-Diamino-2-ethoxyacridinium 2-hydroxybenzoate (compound **3**).

6,9-Diamino-2-ethoxyacridine-DL-lactate monohydrate (0.40 g, 1.107 mmol) and.

2-hydroxybenzoic acid (0.15 g, 1.086 mmol) were gently ground together with 20 drops (about 0.8 ml) of ethanol two times, then treated in an oven at 80 °C for 2 h (**3**). ^1^H NMR (500 MHz, DMSO-d6) δ 12.86 (s, 1H, COOH or OH), 9.01 (s, 2H, NH_2_), 8.25 (d, *J* = 9.2 Hz, 1H, H4), 7.87 (d, *J* = 2.5 Hz, 1H, H5), 7.67 (m, 2H, H8 and H25), 7.53 (dd, *J* = 9.2, 2.5 Hz, 1H, H7), 7.12 (t, *J* = 8.5 Hz, 1H, H23), 6.88 (dd, *J* = 9.2, 2.3 Hz, 1H, H3), 6.84 (s, 2H, NH_2_), 6.62 (d, *J* = 2.3 Hz, 1H, H1), 6.61 (d, *J* = 8.5 Hz, 1H, H22), 6.58 (t, *J* = 7.3 Hz, 1H, H24), 4.16 (q, *J* = 6.9 Hz, 2H, CH_2_), 1.40 (t, *J* = 6.9 Hz, 3H, CH_3_) (Fig. 1S). ^13^C NMR (126 MHz, DMSO-d6) δ 171.84 (C=O), 163.50 (C21), 155.25 (C2), 154.56 (C9), 142.33 (C6), 134.88 (C12 or C14), 131.68 (C12 or C14), 130.37 (C25), 126.37 (C4), 126.33 (C7), 121.11 (C20), 120.08 (C8), 116.41 (C3), 116.23 (C24), 116.21 (C22), 112.35 (C13), 104.66 (C5), 103.37 (C11), 94.68 (C1), 64.44 (CH_2_), 14.99 (CH_3_) (Fig. 2S). ATR-FTIR: 3486 and 3391 cm^−1^ (ν_as and_ ν_sym_ NH_2_), 3352–2000 cm^−1^ (νOH, νNH^+^/overtone and combination bands, νCH), 1685 cm^−1^ (ν_as_C=O, salicylic acid), 1631–1449 cm^−1^ (νC=C, νC=N, NH_2_ and C–H in-plane bend), 1381 cm^−1^ (ν_sym_C=O, salicylic acid), 1324 (OH in-plane bend), 1235–1045 cm^−1^ (νC–N and νC–O), 942 cm^−1^ (OH out-of plane bend), 768–708 cm^−1^ (=C–H out-of-plane bend), 658 cm^−1^ (in-plane ring deformation) (Fig. 3).

d) 6,9-Diamino-2-ethoxyacridinium 2-acetoxybenzoate (compound **4**).

6,9-Diamino-2-ethoxyacridine-DL-lactate monohydrate (0.40 g, 1.107 mmol) and 2-acetoxybenzoic acid (0.2 g, 1.110 mmol) were gently ground together with 20 drops (about 0.8 ml) of ethanol two times, then treated in an oven at 80 °C for 2 h (**4**).

The same pairs of reagents with equimolar ratios were mixed and put in the oven at 80 °C for two hours to verify the possible formation of new species by thermal route only starting from the mechanical mixtures. Only in the case of the pair 6,9-Diamino-2-ethoxyacridine-DL-lactate monohydrate and 2-hydroxybenzoic was the formation of a new compound observed (**5**).

### Attenuated total reflectance–Fourier transform infrared spectroscopy (ATR–FTIR) measurements

The ATR-FTIR spectra were acquired using a Perkin Elmer Spectrum 3™ instrument (Perkin Elmer, Waltham, USA) equipped with attenuated total reflectance (ATR) accessory. The spectra were recorded on the samples without any preparation at room temperature in the spectral range from 4000 to 500 cm^−1^ at a resolution of 4 cm^−1^ averaging 16 scans for each measurement. The FTIR spectra were processed and referred to their baseline using PerkinElmer Spectrum IR Version 10.7.2 software.

### Single-crystal (SCXRD) and powder X-ray diffraction (PXRD) measurements

A suitable single crystal was selected and mounted with epoxy glue on top of glass capillaries for the X-ray diffraction experiments. SCXRD data were collected on an Oxford Diffraction Gemini R ULTRA Ruby CCD diffractometer with CuKα (λ = 1.5418 Å) radiation at T = 295(2) K (Table 1). The lattice parameters were obtained by least-squares fit to the optimised setting angles of the reflections collected by means of CrysAlis CCD. Data were reduced using CrysAlis RED software and applying multi-scan absorption corrections^35^.

**Table 1:** Calculated energies for each moiety in the asymmetric unit and lattice energies (all values are intended for the neutral molecules, except in the **3** salt form case, where ionic forms are considered for both counterparts).

Compound id	Energies calculated for each moiety in the asymmetric unit (kJ/mol)				Lattice energy (kJ/mol)
	6,9-diamino-2-ethoxyacridine	2-hydroxybenzoic acid	Water #1	Water #2	
**1**	− 179.0	− 158.9	− 39.4	− 43.2	− 420.4
**2**	− 245.3	− 291.7	− 65.3	–	− 602.3
**3** (molecular form)	− 136.1	− 78.4	–	–	− 214.5
**3** (salt form)	− 229.8	− 137	–	–	− 366.8

PXRD analysis was carried out on a Bruker D8 Advance diffractometer equipped with a Lynx-Eye XE-T linear detector and CuKα (λ = 1.5418 Å) radiation (Table 2S). Instrument’s goniometer radius is set to 280 mm. The tube was set at operating conditions of 40 mA in current and 40 kV in electric potential. The diffractometer was at first used as a qualitative tool, to verify that the reactions in LAG and in heated mechanical mixture had taken place. Measurements in Bragg–Brentano geometry were carried out in a measurement range from 2° to 70° in 2θ with a step-size of 0.02° and an exposure time of 0.1 s per step. The primary optics consisted of automatic diverging slits keeping the irradiated area constant to 10 mm, followed by 2.5° Soller slits. Since the samples intended for PXRD analysis have already undergone grinding during the attempts of mechanochemical reactions, no further pretreatments were required prior to the measurement. The samples were gently placed as they were in polycarbonate sample holders and subsequently measured. The collected patterns were of sufficient quality to attempt structural resolution. For the final structural refinement, the sample was remeasured in parafocusing geometry conditions in the range from 2° to 130° in 2θ with a step-size of 0.01° and an exposure time of 1.1 s per step, with all other instrument parameters kept as previously described.

### Structural resolutions

The structural resolution procedure from SCXRD data was carried out using the SHELX package^36^. The structures of compounds **1** and **2** were solved with direct methods that carried out refinements by full-matrix least-squares on F^2^ using the SHELXL-2017/1 program^36^. All H-atoms bound to O/N-atoms were located on a different Fourier map and refined freely with U_iso_(H) = 1.5/1.2U_eq_(O/N). All H-atoms bound to C-atoms were placed geometrically and refined using a riding model with d_(C–H)_ = 0.93–0.98 Å and U_iso_(H) = 1.2U_eq_(C) (d_(C–H)_ = 0.96 Å and U_iso(_H) = 1.5U_eq_(C) for the methyl groups.

Compound **3** was solved by powder diffraction data exploiting the approach of simulated annealing in real space, performed by EXPO2014^37^. Torsion angles were refined exploiting Topas Academic v7^38^, which was also used for the final structure refinement.

All interactions were calculated using the PLATON program^39^. The following programs were used to prepare the molecular graphics: ORTEPII^40^, PLUTO-78^41^, and Mercury^42^.

### Hirshfeld surface and energy framework calculation

CrystalExplorer 17.5^43^ was exploited for all ab initio calculations of Hirshfeld surfaces, fingerprint plots and energy frameworks. For all structures, both from single crystal and from powders, the calculations were performed in high-resolution settings. The wave functions for each molecule and pairwise interactions for the estimation of the energy framework were calculated using the algorithm integrated in Crystal Explorer: Tonto, with the B3LYP DFT method by employing the 6–31G(d,p) basic set^44^. The cylinder size scale for the representation of the energy framework has been set to 80 and the cut-off energy value has been set to 0 kJ mol^−1^.

After the pairwise calculation of all interaction energies between the molecules in the asymmetric unit, the lattice energy for each molecule was obtained as arithmetic average of the product of the number of symmetrically equivalent molecules in the cluster, and the total lattice energy (Table 1) was calculated as described in Thomas et al.^45^. Mercury 2022.1 CSD release was used to assess the presence of voids^42^.

## Results and discussion

### Preparation and crystal structure solutions and packing description

The preparation and interconversion of the compounds arising from the combination of salicylic acid derivatives and ethacridine was explored using the three preparation methods reducing solvent amount (solution crystallisation, LAG, and dry grinding/heating) on one hand, to assess how the hydration degree and the preparation method can drive the synthesis toward each specific compound and, on the other hand, to find the process allowing the higher yields, estimated by XRPD quantitative analysis. While Fig. 1 shows the reactants, Fig. 2 summarises the six theoretical combinations of ethacridine with salicylic and acetylsalicylic acid (**1**–**6**), highlighting with the continuous lines the observed and stable products (**1**–**3** ) and the metastable compound **5**, while dashed lines corresponds to the not obtained ones. Interestingly, **3** is very stable and can be obtained starting from both salicylic and acetyl salicylic acid by LAG. **1** and **3** showed a 1:1 ratio but respectively in the dehydrated and anhydrous form. Conversely, acetylsalicylic acid gave only one structure from solution (**2** in 1:1 ratio), while LAG caused hydrolysis and formation of the same compound obtained by LAG starting from ethacridine with salicylic acid.

Compound **4** was not accessible since LAG causes hydrolysis of acetylsalicylic acid before any possible complex formation a **6** was not obtained since dry grinding/heating of acetylsalicylic acid and ethacridine gave no reaction. The three stable products were fully characterized and the corresponding crystal structures were obtained (**1**, **2** and **3**), while compound **5** can be obtained only as a multiphase sample and in low amounts, irrespective of preparation conditions. It was stable enough to obtain a powder diffraction pattern but impossible to prepare as pure or almost pure phase, suitable for crystal structure solution. **5** is included in Fig. 2 to warn when possible metastable phases can be obtained as impurities. Interestingly, aging (in sealed vials or exposed to air moisture) does not induce changes and conversion among different co-crystal forms. The amount of water seems important only during preparation only to drive toward one specific form.

The preparation and crystal structure solution of compound **1**, **2** and **3** is reported in the following sections, and a brief hint to compound **5** is given together with the proof of the impossibility of obtaining compound **4** and **6**. Afterward the comparison of the crystal structures also with support of Hirshfeld surface analysis, and of their energetic features by energy framework calculations is discussed.

### ATR–FTIR analysis

The ATR-FTIR spectra are displayed in Fig. 3 and the details are given in Experimental section. The ATR-FTIR spectra show characteristic vibrational peaks at ~ 3480 and ~ 3370 cm^−1^ assigned to asymmetric and symmetric stretching vibrations of amine groups. A broad and sharp band in the range of ~ 3300–2000 cm^−1^ is caused primarily by the O–H stretching vibrations that obscure the C-H stretching. However, it should be emphasized that the amine salts (NH^+^) have also characteristic absorption bands in this region. The C=O (COO^−^) asymmetric and symmetric stretching of salicylic acid or acetylsalicylic acid were assigned to IR peaks observed at ~ 1670 cm^−1^ and 1380 cm^−1^, respectively^46,47^. The band at 1730 cm^−1^ in the FTIR spectrum of MECHM is attributed to the carbonyl group of lactic acid. Surprisingly, no carbonyl ester stretching vibration was noticed in the FTIR spectrum of compound **2**. However, its presence was confirmed by NMR analysis (vide supra). The IR peaks observed in the range of 1630–1450 cm^−1^ can be assigned to aromatic ring vibrations. However, the appearance of bending vibrations of NH_2_ groups cannot be ruled out in this region of the IR spectra. Moreover, the band at ~ 1450 cm^−1^ may be affected by the asymmetric stretching absorption of CH_3_ group. The vibrational peaks appeared at 1239–1031 cm^−1^ are attributed to C-O and C-N stretching vibrations. The vibrational peaks at 759–669 cm^−1^ were assigned to =C–H bending, whereas the peak at ~ 660 cm^−1^ to in-plane ring deformation.

### Crystal structures description

**Figure 4:**
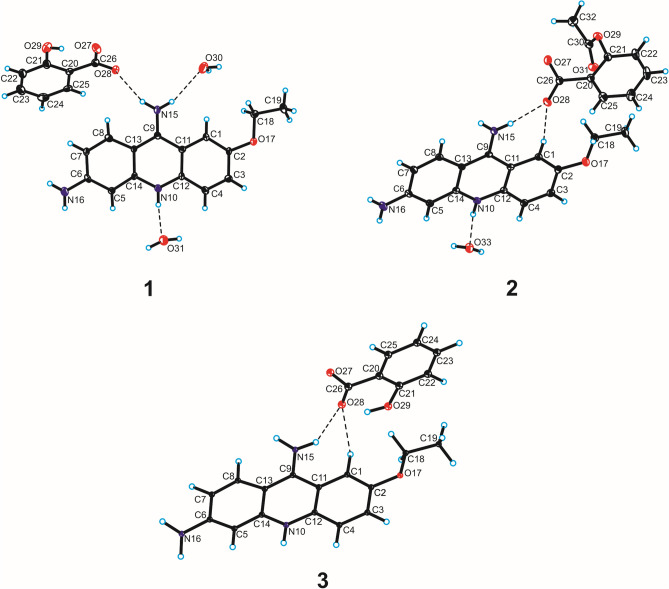
Molecular structure of compounds **1**–**3** showing the atom-labelling scheme. Displacement ellipsoids are drawn at the 25% probability level and H atoms are shown as small spheres of arbitrary radius (hydrogen bonds are represented by dashed line).

The Single-Crystal X-Ray Diffraction measurements show that compound **1** crystallizes in the monoclinic *P*2_1_/c space group with one ethacridine cation, one salicylic acid anion and two water molecules in the asymmetric unit (Fig. 4). In the crystal of compound **1**, the endocyclic N-atom of ethoxyacridinium cation interact with one water molecule by N_(acridine)_–H···O_(water)_ hydrogen bond, whereas amino group in the position 9 of acridine skeleton is linked with the salicylate anion by N_(9-amino)_–H···O_(carboxy)_ hydrogen bond and with a second water molecule through N_(9-amino)_–H···O_(water)_ hydrogen bond. Additionally, the O_(water)_–H···O_(water)_ and O_(water)_–H···O_(carboxy)_ hydrogen bonds occur and, as the consequence, the layers along *c*-axis are observed. The neighbouring layers are linked via O_(water)_–H···O_(carboxy)_ hydrogen bond and π_(acridine)_–π_(acridine)_ interactions building blocks along [0 1 0] direction. In these blocks the π–stacked columns of ethacridinium cation occur. The adjacent columns are connected by N_(6-amino)_–H···O_(hydroxyl)_ hydrogen bonds involving the amino group in position 6 of acridine skeleton and the hydroxyl group of the salicylate anion and create supramolecular cyclic synthons [⋯H–N–H⋯O⋯]_2_ (the 8-membered ring) (Fig. 4). The neighbouring antiparallel columns are also connected by C_(acridine)_–H···π_(salicylate)_ interactions to form a three-dimensional framework structure (Fig. 4).

**Figure 5:**
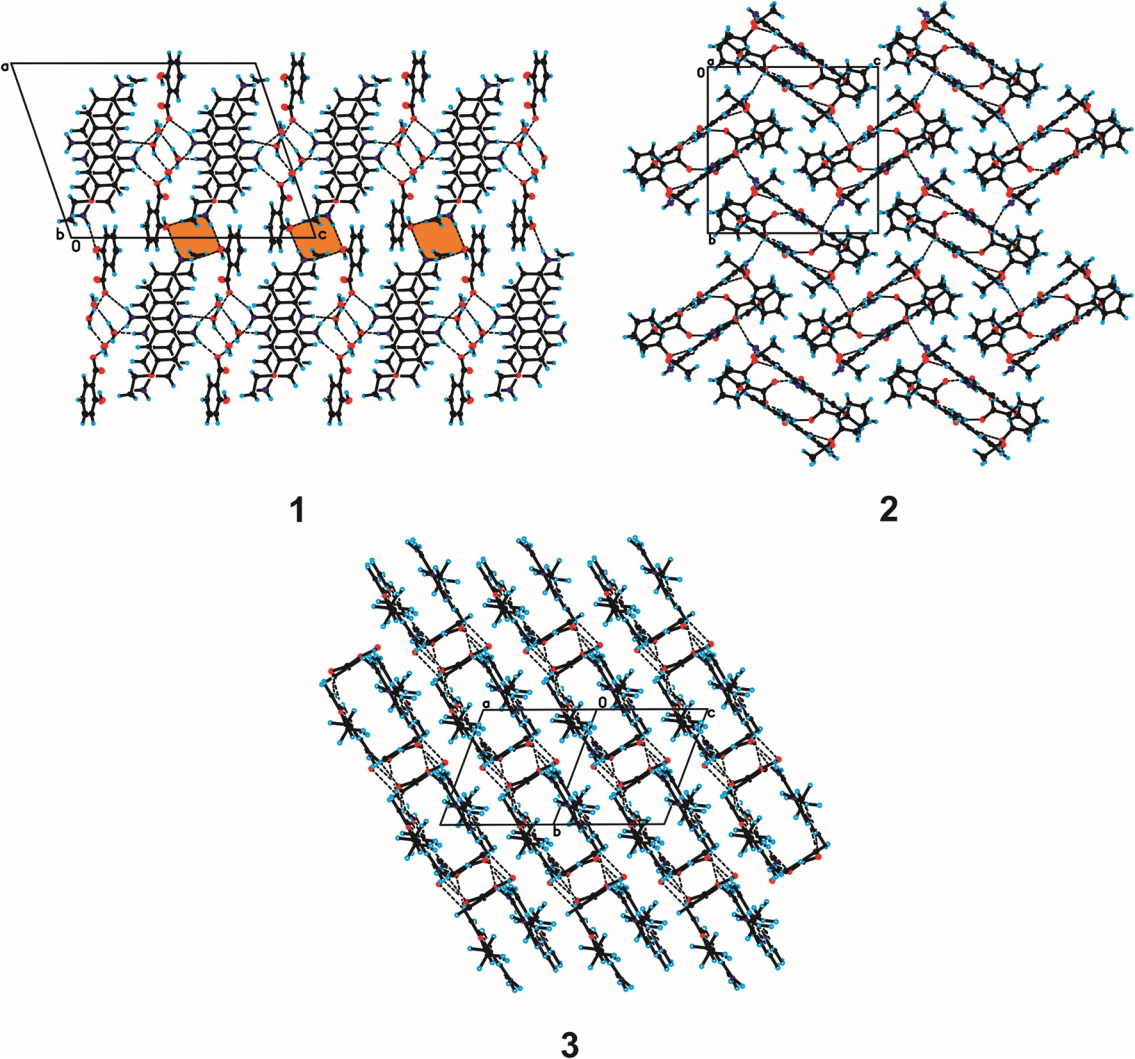
Crystal packing of compounds **1**–**3** (the supramolecular synthon [⋯H–N–H⋯O⋯]_2_ in the crystal packing of compound **1** is highlighted in orange; hydrogen bonds are represented by dashed lines).

The SCXRD measurements show that compound **2** crystallizes in the monoclinic *P*2_1_/n space group with one 6,9-diamino-2-ethoxyacridinium cation, one acetylsalicylic acid anion and one water molecule in the asymmetric unit (Fig. 5). In the crystal packing of compound **2**, the endocyclic N-atom of ethoxyacridinium cation are linked with water molecule by N_(acridine)_–H···O_(water)_ hydrogen bond. In turn, the amino group in the position 9 of acridine skeleton interact with acetylsalicylate anion by N_(9-amino)_–H···O_(carboxy)_ hydrogen bonds to produce supramolecular cyclic synthons [⋯H–N–H⋯(O–C–O)^−^⋯]_2_ (the 12-membered ring) supported by the O_(water)_–H···O_(carboxy)_ hydrogen bonds to create blocks along *a*-axis (Fig. 5). Nevertheless, we do not observe the formation of π–stacked columns of ethoxyacridinium cations. Within the blocks, the 6,9-diamino-2-etoxyacridinium cations interact by π_(acridine)_–π_(acridine)_ interactions, whereas cations interact with anions through the N_(6-amino)_–H···O_(carbonyl)_ and C_(acridine)_–H···O_(acetylsalicylate)_ hydrogen bonds between cation and anion. The neighbouring blocks are arranged in a herringbone pattern and are connected via one N_(6-amino)_–H···O_(carboxy)_ hydrogen bond to create a three-dimensional framework structure (Fig. 5).

**Figure 6:**
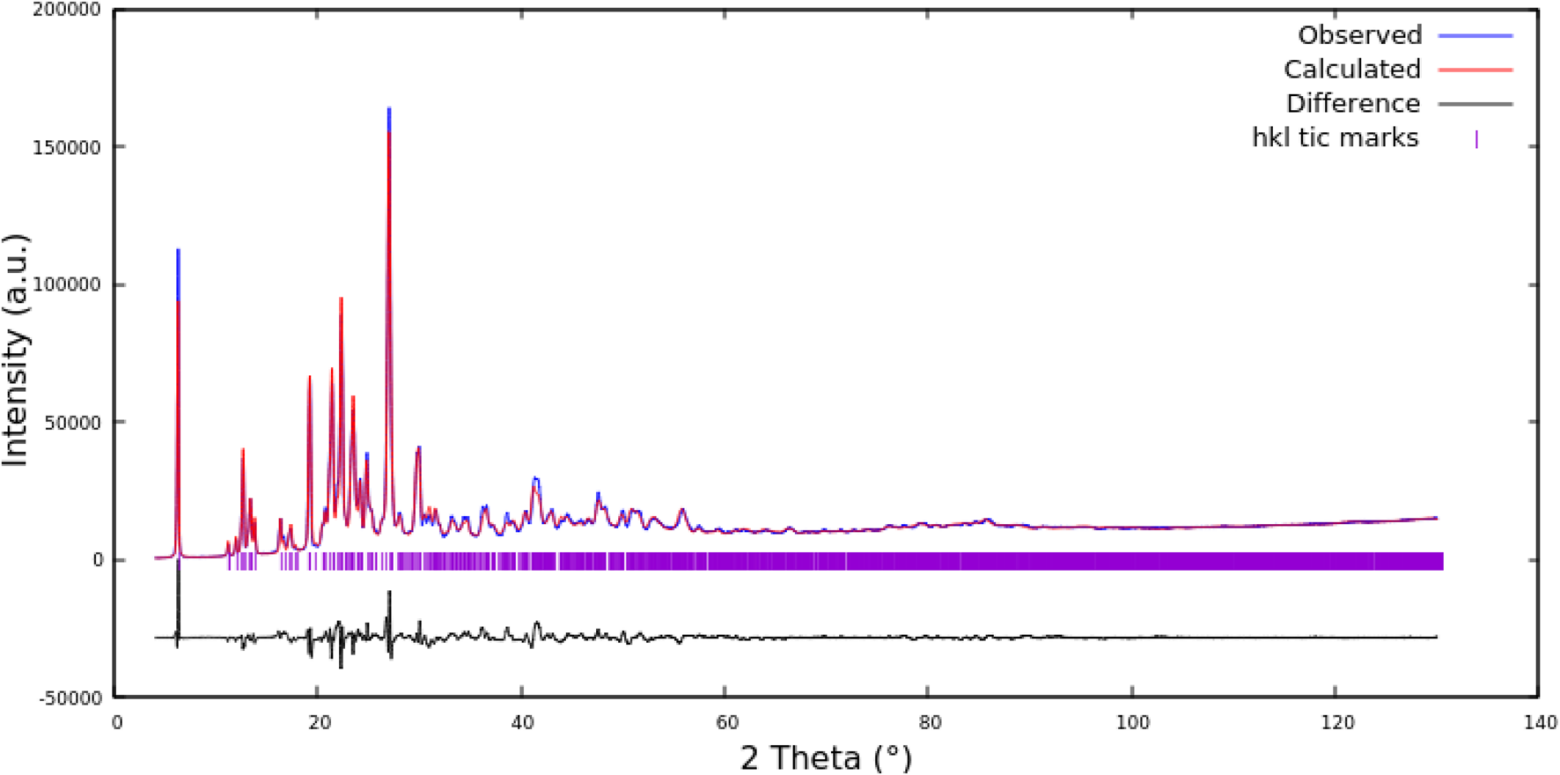
Rietveld refinement of compound **3** PXRD pattern.

The PXRD measurements (Fig. 6) show that compound **3** crystallizes in an anhydrous form in the triclinic *P*-1 space group with one 6,9-diamino-2-ethoxyacridinium cation and one acetylsalicylic acid anion in the asymmetric unit (Fig. 4). In the crystal of compounds **3**, due to the absence of water molecule the one O-atom from the carboxyl group of salicylate anion is linked with endocyclic N-atom of ethoxyacridinium cation through N_(acridine)_–H···O_(carboxy)_ hydrogen bond, whereas second O-atom are connected with amino group in the position 9 of acridine skeleton by N_(9-amino)_–H···O_(carboxy)_ hydrogen bond, therefore, cations and anions produce blocks along [1 0 1] direction (Fig. 6). In these blocks, the π_(acridine)_–π_(acridine)_ interactions and C_(acridine)_–H···O_(salicylate)_ hydrogen bonds also occur, but the π–stacked columns of ethoxyacridinium cations are not observed, like for compound **2**. The neighbouring related by the center of symmetry columns are connected by N_(9-amino)_–H···O_(carboxy)_ and C_(acridine)_–H···O_(salicylate)_ hydrogen bonds to form a 3-D framework structure (Fig. 6).

An analysis of the crystal packing of compounds **1**–**3** shows that in the crystal of compound **3** the ions are packed more efficiently than in the crystals of the compounds **1** and **2**. Compound **3** have a significantly higher Kitaigorodskii type of packing index with the percentage of filled space equal to 73.4% (67.7% and 70.7% for compound **1** and **2**, respectively) and crystal density equal to 1.398 g/cm^3^ (1.377 g/cm^3^ and 1.330 g/cm^3^, for compound **1** and **2**, respectively) than other compounds. This is due to the absence of a water molecule in the crystal lattice. Moreover, the Rietveld refinement of XRPD pattern of **3** allowed obtaining the quantitative phase analysis indicating residuals below 0.2% of reactants. Since the LAG process avoids wastes, and also considering possible error in estimating phase amounts by XRPD, this means that the yield of compound **3** preparation is at least larger than 99%. Conversely, when exploiting solution crystallization, large number of reactants are obtained mixed with single crystals of **1** and **2**.

### Unstable and metastable combinations

In this section, the absence of compound **4** and **6** and the elusive appearance of compound **5** is discussed. When carrying out the LAG synthesis between acetylsalicylic acid and ethacridine instead of obtaining compound **4**, the reaction product is once again compound **3**, which is obtained following a hydrolysis which removes the acetyl from the aromatic ring. This is a further proof of the high stability of compound **3**. Dry grinding/thermal treatment of acetylsalicylic acid and ethacridine gave no reaction, probably because of the steric hindrance of acetyl side chain towards the reaction with the more planar ethacridine.

**Figure 7:**
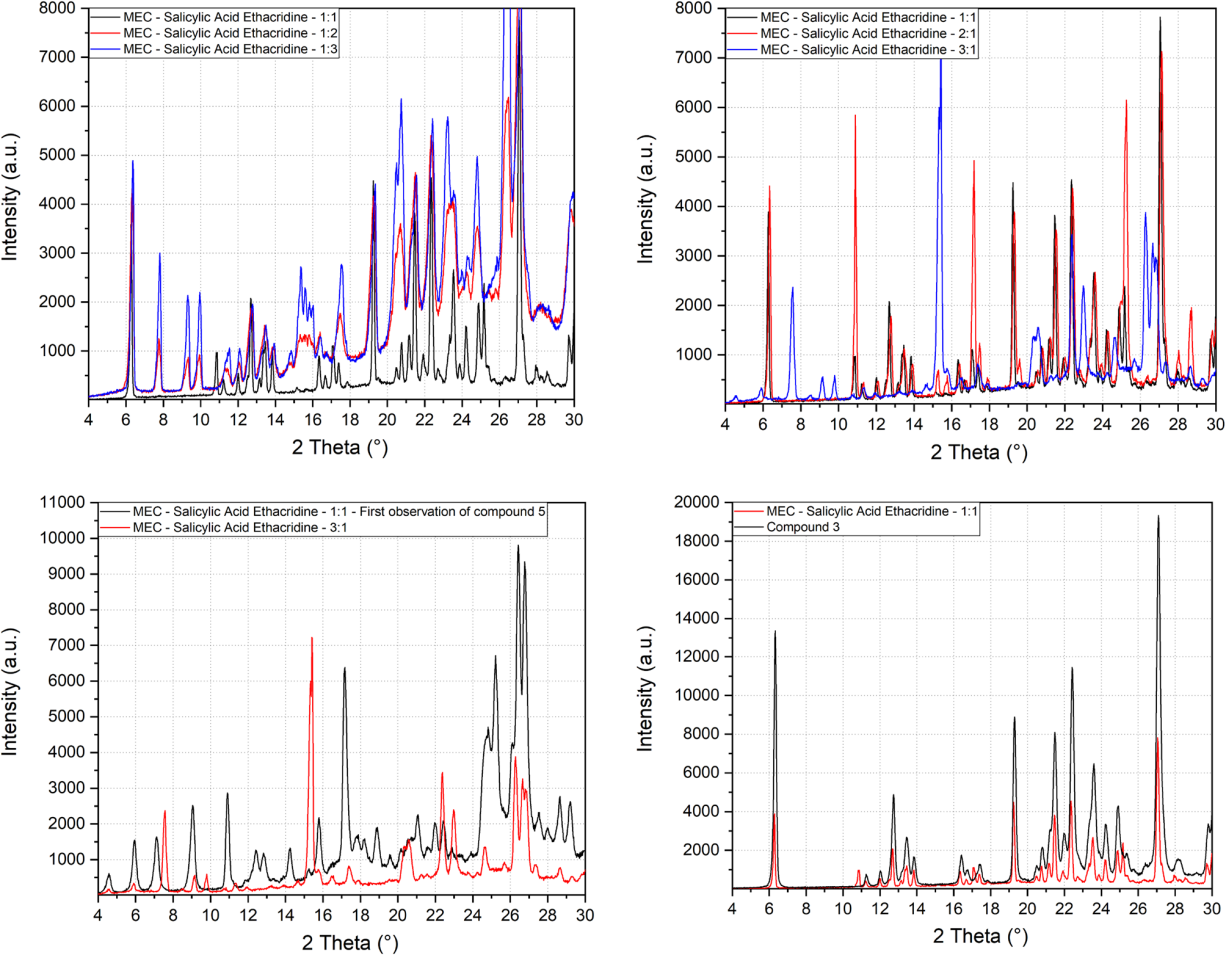
Comparison of PXRD pattern of compounds **3** and **5** with the corresponding mechanical mixtures at different ratios.

Conversely, a new phase was obtained from the thermal treatment of the mechanical mixture of ethacridine and salicylic acid (compound **5**). In Fig. 7 it can be seen how the new phase is mixed with the reagents, which has made the cell indexing impossible. Attempts were made to purify the new phase with a set of experiments in which the molar ratios of the two reagents were changed. This was done to identify crystals with a ratio other than 1:1 between salicylic acid and ethacridine more easily. Samples were then prepared with different molar ratios, from 3:1 to 1:3 passing through all the integer intermediates. Only in two samples (Fig. 7c) it is possible to observe the presence of the new phase, of which the peak at 4.5° in 2theta is characteristic. However, in both samples the presence of reagents is too high for an attempt at indexing the cell. In all the other samples, the presence of compound **3** can be observed, an indication of the fact that the one obtained by simple aging of the physical mixture, without grinding, is a less stable phase than the one isolated by a mechanochemical process.

### Hirshfeld surface and energy framework calculations

Hirshfeld surface analysis, performed on the refined structure models of the compounds, allows to highlight the surroundings and interactions of each molecule in the packing by a visual schematic representation of the contact distances called the “fingerprint plot”. In the fingerprint plot, the Hirshfeld surface is made bidimensional by plotting, for each pixel of the surface, the distance from the nearest point of the moiety inside the surface (di) versus the distance from the nearest point of the moiety outside the surface (de). In this plot, spikes extending toward the origin of the axes indicate the shortest contacts. The colour of each point corresponds to the area of the surface with that combination of de and di. The colour scale goes from blue for the pairs of coordinates that occur less frequently, thus contributing minimally to the surface, to green and then red for the most frequent ones. The atoms involved in the contact can be deduced by applying elemental filters on the fingerprint plot.

The fingerprint plots obtained from Hirshfeld surface calculations on compound **1** (Fig. 14S) confirm that the main interactions driving the packing are H⋅⋅⋅O hydrogen bonds and π-stacking (C⋅⋅⋅C) interactions between the 6,9-diamino-2-ethoxyacridinium moieties with an average C⋅⋅⋅C distance of 3.6 Å.

The hydrogen bonds form a complex net connecting all four moieties in the asymmetric unit reciprocally, as represented by the coloured lines in Fig. 14S.

Regarding compound **2**, the fingerprint plots calculated from the Hirshfeld surfaces of the moieties confirm that the main interactions are the two hydrogen bonds that the water molecules form bridging between the 6,9-diamino-2-ethoxyacridinium cation and the 2-acetoxybenzoate anion and the π-stacking interactions between 6,9-diamino-2-ethoxyacridinium moieties with an average C⋅⋅⋅C distance of about 3.6 Å which are also highlighted by the alternated red and blue triangles visible on the Hirshfeld surface with the Shape index plotted (Fig. 15Sd).

**Figure 8:**
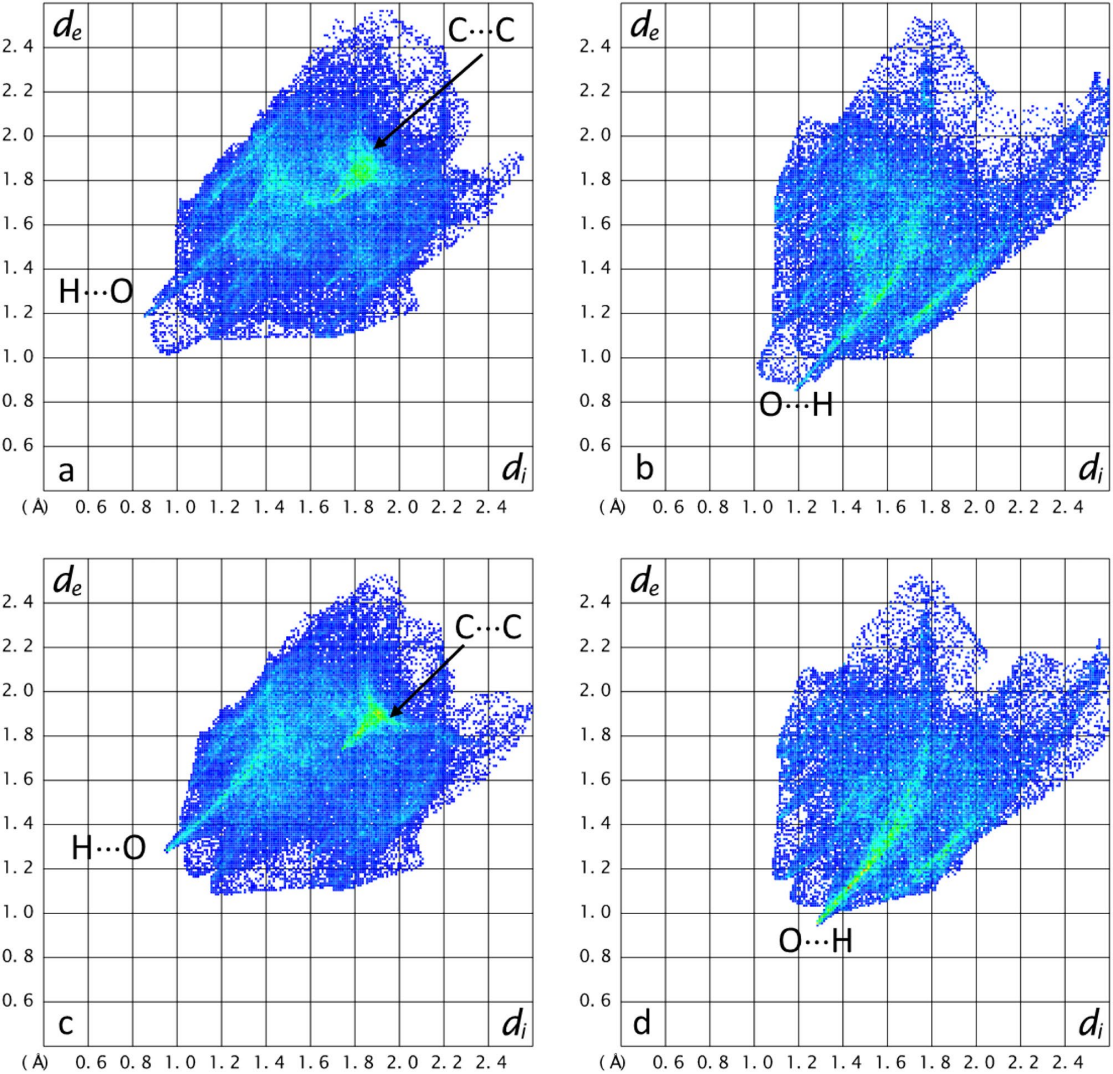
Fingerprint plots for compound** 3** in the molecular (**a**, **b**) and ionic form (**c**, **d**). In the first row: (**a**) 6,9-diamino-2-ethoxyacridine (**b**) 2-hydroxybenzoic acid from the molecular form of compound **3**. In the second row: (**c**) 6,9-diamino-2-ethoxyacridinium and (**d**) 2-hydroxybenzoate from the salt form of compound **3**.

**Figure 9:**
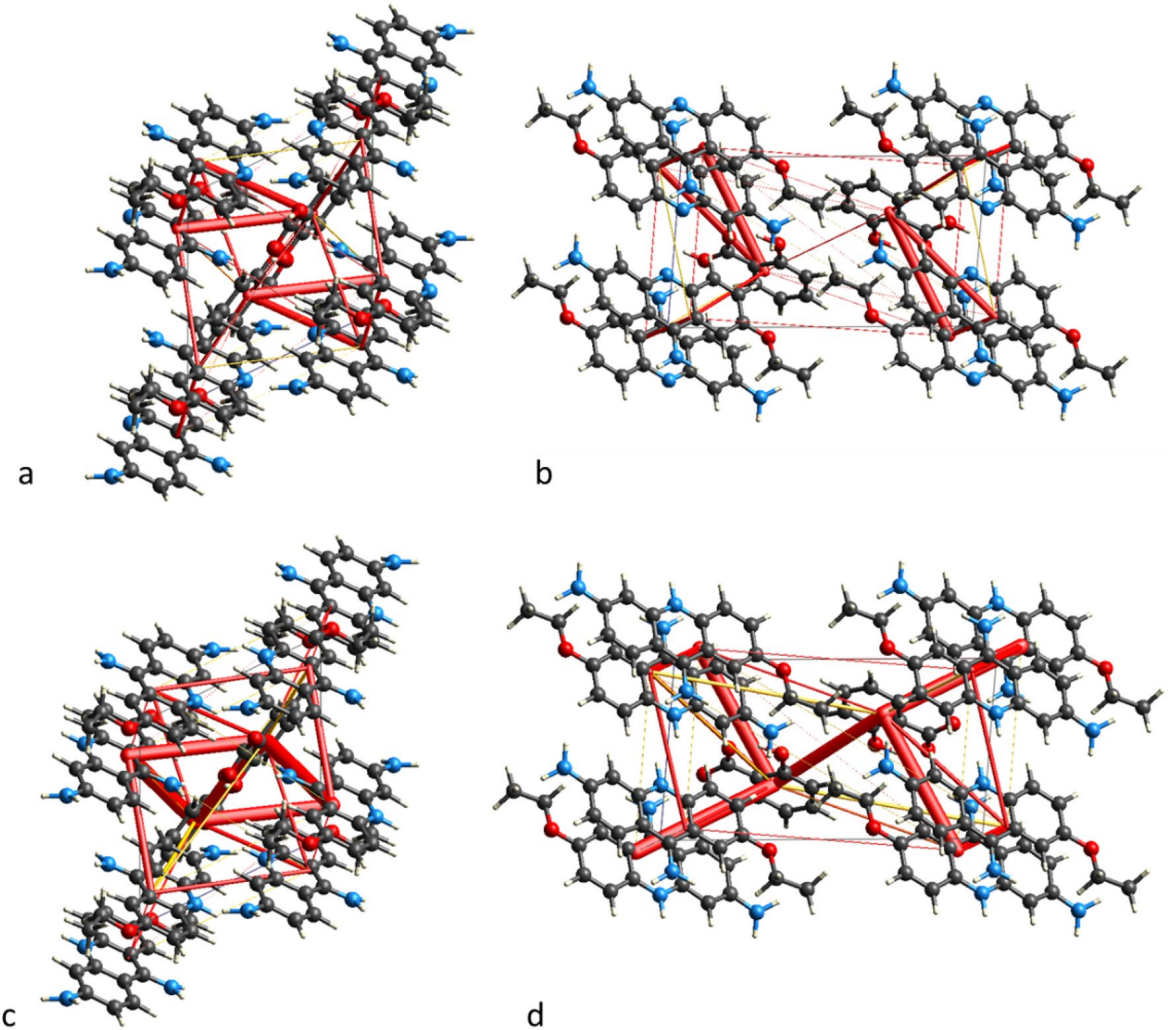
Energy frameworks for compound **3**: Coulomb interactions in the molecular (**a**, **b**) and salt (**c**, **d**) form, viewed along *a*-axis and *b*-axis respectively. The interactions are depicted as rods of thickness proportional to the strength of the interaction. Attractive forces are represented by red rods and the repulsive ones by yellow rods.

Hirshfeld surface analysis was performed on both the molecular and salt form of compound **3** to verify the position of the hydrogen atom from the viewpoint of structural stability. The surfaces and relevant distances in the two forms are reported in Fig. 13S in the ESI file. In Fig. 9 the fingerprint plots for the molecular form and the salt form of the compound are reported for each moiety in the crystal. The main interactions are π-stacking interactions between the aromatic ring of 6,9-diamino-2-ethoxyacridine/ium moieties and hydrogen bonds between 6,9-diamino-2-ethoxyacridine/ium and 2-hydroxybenzoate/benzoic acid. By looking at the filtered fingerprint plots the amount of surface involved in H⋅⋅⋅O interactions is larger in the salt form: 15.9% (including reciprocal contacts) (Fig. 8c) and 32.4% (Fig. 8d) versus 11.7% (Fig. 8a) and 22% (Fig. 8b) in the molecular form. This means that, while the H⋅⋅⋅O contacts are longer in the salt form, they are more directional, therefore the typical spike feature in the fingerprint plot appears more definite and in green colour.

The pairwise interaction energies and lattice energies were calculated, and the results are reported in Table 1. The resulting energies show that the presence of water stabilizes the structure by creating hydrogen bonds and lowering the overall lattice energy, since the energies of compounds **1** and **2** are lower with respect to compound **3**.

Energy frameworks were plotted for compound **3** in both the molecular and ionic form (Table 1), and the representation of the coulombic forces in the framework is presented in Fig. 9. The large gap in energy between the two forms (Table 1) strongly confirms that the compound is in salt form since the structure can accommodate the ionic bond with a very favorable ratio between the attractive forces (red rods) and the repulsion ones (yellow rods).

## Conclusions

Four new compounds were obtained combining ethacridine and salicylic and acetylsalicylic acid by different preparation methods, and three out of them were stable enough to determine their structures by X-ray diffraction. Two structures (**1** and **3**) were obtained combining ethacridine with salicylic acid by solution and LAG respectively. They have the same 1:1 ratio but different hydration degree. The crystal structure of **3** was solved by PXRD since suitable single crystal could not be obtained. Hirshfeld surface analysis and energy framework calculations confirmed, with a tool independent from powder diffraction, that **3** is a anhydrous salt while **1** is a dihydrated molecular crystal. A third ethacridine/salicylic metastable phase was identified by powder diffraction, but its appearance was elusive and not reproducible, and it was impossible obtaining its crystal structure. Conversely, when combining ethacridine and acetylsalicylic acid, only one compound (in 1:1 ratio and monohydrated) was obtained from solution (**2**), while LAG caused hydrolysis and formation of the same compound obtained by LAG of the ethacridine/salicylic acid mixture. Interestingly, solution precipitation gave dihydrated (**1**) or monohydrated (**2**) structures with low yields, while LAG allowed obtaining an anhydrous salicylic acid/ethacridine salt complex (**3**) with a yield close to 1, much larger than those obtained by solution crystallization, confirming that LAG can drive the synthesis toward the more stable form. Moreover, the synthesis methods also influenced the final crystal packing, since the two structures obtained by solution crystallizations maximize π_(acridine)_–π_(acridine)_ contacts with a less compact packing, while the LAG structure is more compact with a packing driven by hydrogen bonds, as confirmed by Hirshfeld analysis.

### Supplementary Information

Supplementary Information.

## Data Availability

Full crystallographic details the structures reported in this paper have been deposited with the Cambridge Crystallographic Data Centre (deposition No. CCDC 2279198, CCDC 2279199 and CCDC 2280612 for **1**, **2** and **3** respectively) and they may be obtained from www: http://www.ccdc.cam.ac.uk, e-mail: deposit@ccdc.cam.ac.uk or The Director, CCDC, 12 Union Road, Cambridge, CB2 1EZ, UK. The datasets used and/or analyzed during the current study are available from the corresponding author upon reasonable request.
