# Supplementary material for: Crystallization from solution versus mechanochemistry to obtain double-drug multicomponent crystals of ethacridine with salicylic/acetylsalicylic acids
Source: Sci Rep. 2024 Jan 21;14:1834. doi: 10.1038/s41598-023-49922-4 (PMC10800331; doi:10.1038/s41598-023-49922-4)
Supplement: Supplementary file 1 — Supplementary Information. [file 41598_2023_49922_MOESM1_ESM.pdf]

Electronic Supplementary Information

Crystallization from solution versus mechanochemistry to obtain double-drug multicomponent crystals of ethacridine with salicylic/acetylsalicylic acids

Artur Mirocki,[a] Mattia Lopresti,[b] Luca Palin,[b,c] Eleonora Conterosito,[d] Emilia Sikorska,[a] Artur Sikorski[a] and Marco Milanesio[b]*

[a] *Faculty of Chemistry of the University of Gdansk, ul. Wita Stwosza 63, 80-308 Gdansk, Poland.*

[b] *Università del Piemonte Orientale, Dipartimento di Scienze e Innovazione Tecnologica, Viale T. Michel 11, 15121 Alessandria, Italy.*

[c] *Nova Res s.r.l., Via D. Bello 3, 28100 Novara, Italy.*

[d] *Università del Piemonte Orientale, Dipartimento per lo Sviluppo Sostenibile e la Transizione Ecologica, Viale T. Michel 11Piazza Sant'Eusebio 5, 13100 Vercelli, Italy.*

** Corresponding Author: Marco Milanesio; E-mail: marco.milanesio@uniupo.it*

Synthesis of the compounds

a) 6,9-Diamino-2-ethoxyacridine-DL-lactate monohydrate (0.05 g, 0.138 mmol) and 2-hydroxybenzoic acid (0.011 g, 0.08 mmol) were dissolved in 3 mL of an ethanol/water mixture (2:1 v/v) and heated for 15 min to dissolve the sample. The solution was allowed to evaporate for a few days to give yellow crystals (**1**).

b) 6,9-Diamino-2-ethoxyacridine-DL-lactate monohydrate (0.03 g, 0.083 mmol) and 2-acetoxybenzoic acid (0.015 g, 0.083 mmol) were dissolved in 3 mL of an ethanol/water mixture (2:1 v/v) and heated for 15 min to dissolve the sample. The solution was allowed to evaporate for a few days to give yellow crystals (**2**).

c) 6,9-Diamino-2-ethoxyacridine-DL-lactate monohydrate (0.40 g, 1.107 mmol) and 2-hydroxybenzoic acid (0.15 g, 1.086 mmol) were gently ground together with 20 drops (about 0.8 ml) of ethanol two times, then treated in an oven at 80°C for 2 hours (**3**).

d) 6,9-Diamino-2-ethoxyacridine-DL-lactate monohydrate (0.40 g, 1.107 mmol) and 2-acetoxybenzoic acid (0.2 g, 1.110 mmol) were gently ground together with 20 drops (about 0.8 ml) of ethanol two times, then treated in an oven at 80°C for 2 hours (**4**).

For all compounds, crystallization from solutions was carried out from different solvents or mixture of solvents, but crystals of other polymorphs, solvates or compounds with different stoichiometry of the reactants were not observed. It also concerns the LAG synthesis. We do not observe that amount of the liquid added in LAG reactions affect the product outcome.

The same pairs of reagents with equimolar ratios were mixed and put in the oven at 80°C for two hours to verify the possible formation of new species by thermal route only starting from the mechanical mixtures. Only in the case of the pair 6,9-Diamino-2-ethoxyacridine-DL-lactate monohydrate and 2-hydroxybenzoic was the formation of a new compound observed (**5**).

2. Nuclear magnetic resonance (NMR) and Attenuated Total Reflectance – Fourier Transform Infrared Spectroscopy (ATR–FTIR) measurements

The ^{1}H and ^{13}C 1D NMR, 2D ^{1}H-^{1}H COSY and ^{1}H-^{13}C 2D HSQC experiments were conducted at 298 K on a Bruker III Avance 500 MHz spectrometer (^{1}H frequency 500.13 Hz) operated at magnetic fields of 11.7 T. The concentrations of the samples were 0,015 mM in DMSO-d_6. The heteronuclear spectra were recorded on natural abundance of ^{13}C isotope. The proton-proton coupling constants in solution were directly extracted in ^{1}H NMR spectra using the multiplet analysis tool in MestreNova 7.0.1 (Masterlab Research S.L.).

The ^{1}H and ^{13}C NMR spectra of all compounds are displayed in Figures 1S-2S. Considering that they look almost identical, the 2D NMR spectra were recorded only for the compound **1** (Figure 3) to facilitate unambiguous recognition of the NMR signals of protons and protonated carbons. The quaternary carbons were assigned based on the literature data. In addition, the ^{13}C chemical shifts were predicted using the https://www.nmrdb.org service and compared with experimental values. The ^{1}H and ^{13}C NMR data are summarized in the experimental section. Chemical shifts, multiplicities and the observed proton-proton coupling constants are in accordance with the structure of the studied compounds. A broad singlet at ~13 ppm in the ^{1}H NMR spectra indicates either a carboxylic proton or proton from hydroxyl group in salicylic acid, which is strongly deshelled due to intramolecular hydrogen bonding. Resonances between 6.5 and 8.5 ppm correspond to aromatic protons. The protons from both amine groups attached to the acridine ring system are found at ~6.8 and 9 ppm. NMR resonances with chemical shifts less than 4.2 ppm correspond to aliphatic protons of the ethoxy group and lactic acid. The number of carbon signals corresponds to the expected number of carbon atoms of the studied compounds.

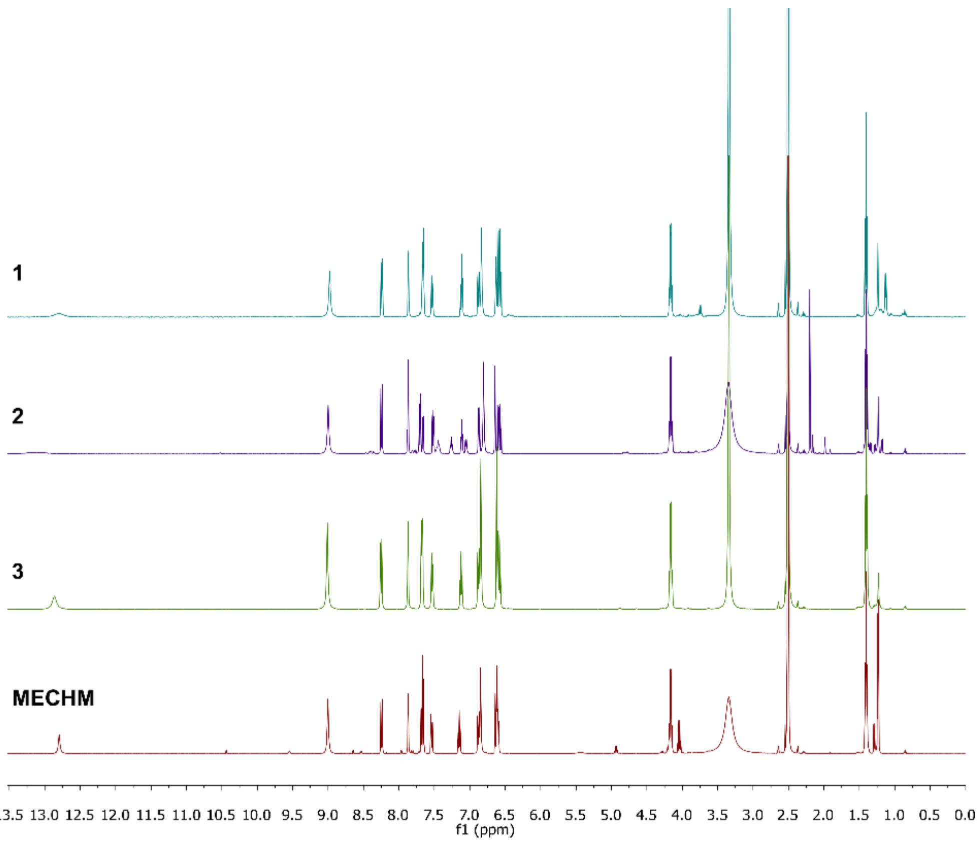

Figure 1S. ^{1}H NMR spectra of studied compounds in DMSO-d_6 at 298 K (MECHM is mechanical mixture of ethacridinium lactate and salicylic acid).

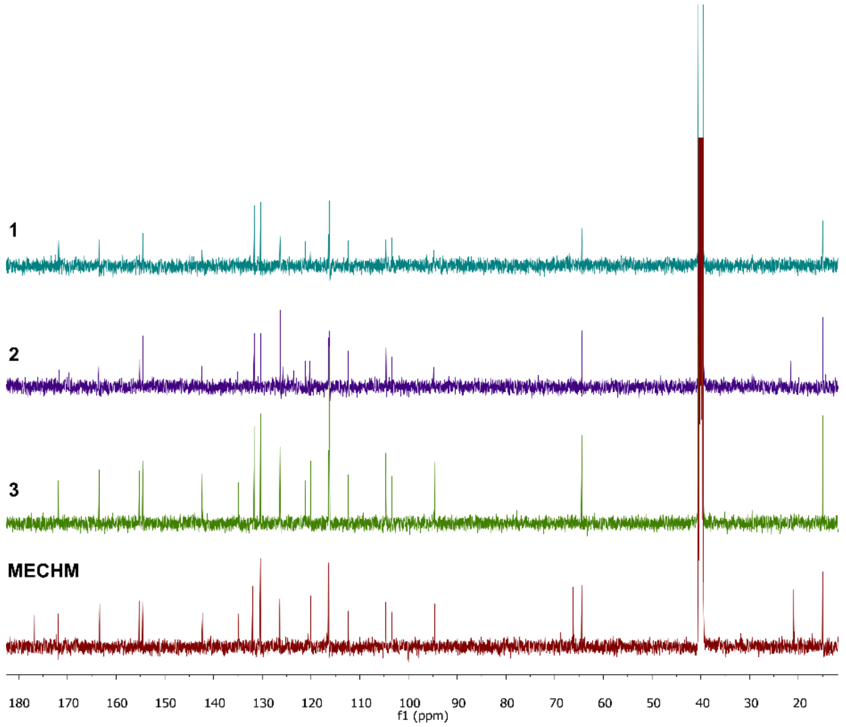

Figure 2S. ^{13}C NMR spectra of studied compounds in DMSO-d_6 at 298 K.

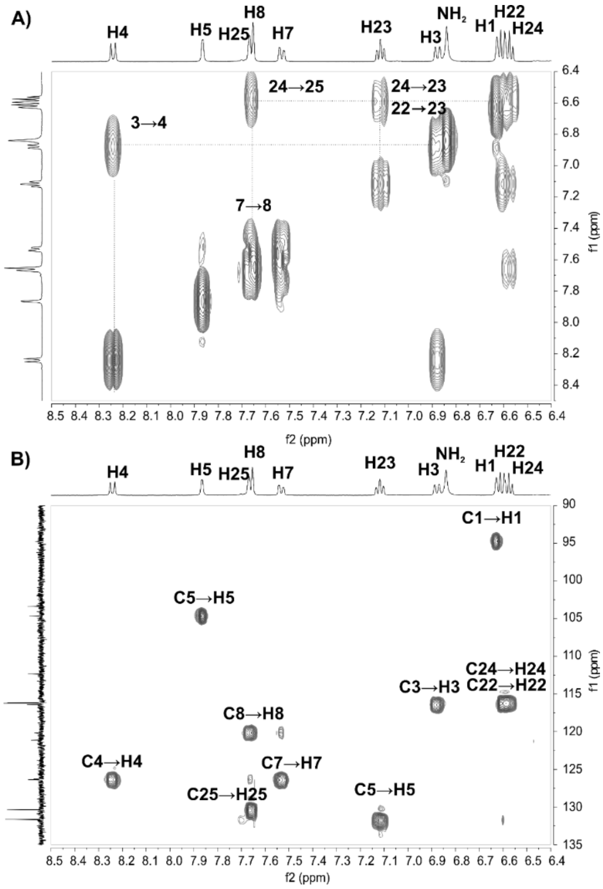

Figure 3S. ^{1}H-^{1}H COSY (a) and ^{1}H-^{13}C HSQC (b) spectra of the compound **1** in DMSO-d$_6$ at 298 K.

Compound 1. ^{1}H NMR (500 MHz, DMSO) δ 12.80 (bs, 1H, COOH or OH), 8.98 (s, 2H, NH_2), 8.24 (d, *J* = 9.2 Hz, 1H, H4), 7.87 (d, *J* = 2.5 Hz, 1H, H5), 7.66 (m, 2H, H8 and H25), 7.53 (dd, *J* = 9.2, 2.5 Hz, 1H, H7), 7.11 (td, *J* = 7.4, 1.8 Hz, 1H, H23), 6.88 (d, *J* = 9.2, 1H, H3), 6.84 (s, 2H, NH_2), 6.63 (s, 1H, H1), 6.60 (d, *J* = 8.2 Hz, 1H, H22), 6.57 (t, *J* = 7.4 Hz, 1H, H24), 4.17 (q, *J* = 6.9 Hz, 2H, CH_2), 1.41 (t, *J* = 6.9 Hz, 3H, CH_3). ^{13}C NMR (126 MHz, DMSO) δ 171.80 (C=O), 163.53 (C21), 155.23 (C2), 154.52 (C9), 142.38 (C6), 134.89 (C12 or C14), 131.69 (C12 or C14), 131.64 (C23), 130.35 (C25), 126.37 (C4), 126.30 (C7), 121.13 (C20), 120.16 (C8), 116.42 (C3), 116.22 (C24), 116.16 (C22), 112.35 (C13), 104.65 (C5), 103.38 (C11), 94.76 (C1), 64.45 (CH_2), 15.00 (CH_3). ATR-FTIR: 3475 and 3364 cm^{-1} ($\nu_{as\ and}\nu_{sym}$ NH_2), 3290 and 2000 cm^{-1} (νOH, νNH^+/overtone and combination bands, νCH), 1678 cm^{-1} (shoulder band, ν_{as}C=O salicylic acid), 1621-1453 cm^{-1} (νC=C, νC=N, NH_2 and C-H in-plane bend), 1381 cm^{-1} (ν_{sym}C=O, salicylic acid), 1333 (OH in-plane bend), 1238-1031 cm^{-1} (νC-N and νC-O), 759-706 cm^{-1} (=C-H out-of-plane bend), 664 cm^{-1} (in-plane ring deformation).

Compound 2. ^{1}H NMR (500 MHz, DMSO) δ 13.11 (bs, 1H, COOH or OH), 9.00 (s, 2H, NH_2), 8.25 (d, *J* = 9.2 Hz, 1H, H4), 7.87 (d, *J* = 2.5 Hz, 1H, H5), 7.70 (d, *J* = 9.2 Hz, 1H, H8), 7.66 (dd, *J* = 7.6, 1.8 Hz, 1H, H25), 7.52 (dd, *J* = 9.2, 2.5 Hz, 1H, H7), 7.11 (td, *J* = 7.6, 1.8 Hz, 1H, H23), 6.87 (dd, *J* = 9.2, 2.1 Hz, 1H, H3), 6.81 (s, 2H, NH_2), 6.64 (d, *J* = 2.1 Hz, 1H, H1), 6.60 (d, *J* = 8.2 Hz, 1H, H22), 6.57 (t, *J* = 7.6 Hz, 1H,

H24), 4.16 (q, *J* = 6.9 Hz, 2H, CH_2), 2.20 (s, 3H, CH_3), 1.40 (t, *J* = 7.0 Hz, 3H). ^{13}C NMR (126 MHz, DMSO) δ 171.73 (C=O), 169.71 (C=O), 163.56 (C21), 155.14 (C2), 154.49 (C9), 142.44 (C6), 135.06 (C12 or C14), 131.70 (C12 or C14), 131.62 (C23), 130.34 (C25), 126.29 (C4), 125.80 (C7), 121.17 (C20), 120.28 (C8), 116.40 (C3), 116.22 (C24), 116.13 (C22), 112.34 (C13), 104.60 (C5), 103.42 (C11), 94.86 (C1), 64.43 (CH_2), 21.60 ($\underline{CH_3}$-C=O), 15.00 (CH_3). ATR-FTIR. 3474 and 3368 cm^{-1} ($ν_{as\ and}\ ν_{sym}$ NH_2), 3356-2000 cm^{-1} (νOH, $νNH^+$/overtone and combination bands, νCH), 1671 cm^{-1} ($ν_{as}$C=O, salicylic acid), 1634-1456 cm^{-1} ($ν_{C=C}$ and $ν_{C=N,}$ NH_2 and C-H in-plane bend), 1381 cm^{-1} ($ν_{sym}$C=O, acetylosalicylic acid), 1326 (OH in-plane bend), 1236-1031 cm^{-1} (νC-N and νC-O), 942 cm^{-1} (OH out-of plane bend), 759-706 cm^{-1} (=C-H out-of-plane bend), 663 cm^{-1} (in-plane ring deformation).

Compound 3. ^1H NMR (500 MHz, DMSO) δ 12.86 (s, 1H, COOH or OH), 9.01 (s, 2H, NH_2), 8.25 (d, *J* = 9.2 Hz, 1H, H4), 7.87 (d, *J* = 2.5 Hz, 1H, H5), 7.67 (m, 2H, H8 and H25), 7.53 (dd, *J* = 9.2, 2.5 Hz, 1H, H7), 7.12 (t, *J* = 8.5 Hz, 1H, H23), 6.88 (dd, *J* = 9.2, 2.3 Hz, 1H, H3), 6.84 (s, 2H, NH_2), 6.62 (d, *J* = 2.3 Hz, 1H, H1), 6.61 (d, *J* = 8.5 Hz, 1H, H22), 6.58 (t, *J* = 7.3 Hz, 1H, H24), 4.16 (q, *J* = 6.9 Hz, 2H, CH_2), 1.40 (t, *J* = 6.9 Hz, 3H, CH_3). ^{13}C NMR (126 MHz, DMSO) δ 171.84 (C=O), 163.50 (C21), 155.25 (C2), 154.56 (C9), 142.33 (C6), 134.88 (C12 or C14), 131.68 (C12 or C14), 130.37 (C25), 126.37 (C4), 126.33 (C7), 121.11 (C20), 120.08 (C8), 116.41 (C3), 116.23 (C24), 116.21 (C22), 112.35 (C13), 104.66 (C5), 103.37 (C11), 94.68 (C1), 64.44 (CH_2), 14.99 (CH_3). ATR-FTIR: 3486 and 3391 cm^{-1} ($ν_{as\ and}\ ν_{sym}$ NH_2), 3352-2000 cm^{-1} (νOH, $νNH^+$/ overtone and combination bands, νCH), 1685 cm^{-1} ($ν_{as}$C=O, salicylic acid), 1631-1449 cm^{-1} (νC=C, νC=N, NH_2 and C-H in-plane bend), 1381 cm^{-1} ($ν_{sym}$C=O, salicylic acid), 1324 (OH in-plane bend), 1235-1045 cm^{-1} (νC-N and νC-O), 942 cm^{-1} (OH out-of plane bend), 768-708 cm^{-1} (=C-H out-of-plane bend), 658 cm^{-1} (in-plane ring deformation).

Mechanical mixture. ^1H NMR (500 MHz, DMSO): δ 12.80 (s, 1H, COOH or OH), 9.00 (s, 2H, NH_2), 8.25 (d, *J* = 9.2 Hz, 1H, H4), 7.87 (d, *J* = 2.5 Hz, 1H, H5), 7.67 (m, 2H, H8 and H25), 7.54 (dd, *J* = 9.2, 2.5 Hz, 1H, H7), 7.15 (td, *J* = 7.6, 1.8 Hz, 1H, H23), 6.88 (dd, *J* = 9.2, 2.1 Hz, 1H, H3), 6.85 (s, 2H, NH_2), 6.64 (s, 1H, H1), 6.61 (m, 5H, H22 and H24), 4.17 (q, *J* = 6.9 Hz, 2H, CH_2), 4.04 (q, *J* = 6.9 Hz, 1H, CH), 1.40 (t, *J* = 6.9 Hz, 3H, CH_3), 1.24 (d, *J* = 6.9 Hz, 3H, CH_3). ^{13}C NMR (126 MHz, DMSO) δ 176.76 (C=O), 171.85 (C=O), 163.37 (C21), 155.28 (C2), 154.58 (C9), 142.30 (C6), 134.83 (C12 or C14), 132.06 (C12 or C14), 131.99 (C23), 130.39 (C25), 126.40 (C4), 126.33 (C7), 120.55 (C20), 120.04 (C8), 116.45 (C3), 116.41 (C24), 116.33 (C22), 112.35 (C13), 104.68 (C5), 103.35 (C11), 94.63 (C1), 66.22 (CH), 64.45 (CH_2), 20.88 (CH_3), 14.99 (CH_3). ATR-FTIR: 3474 and 3366 cm^{-1} ($ν_{as\ and}\ ν_{sym}$ NH_2), 3300-2000 cm^{-1} (νOH, $νNH^+$/ overtone and combination bands, νCH), 1730 cm^{-1} (νC=O, lactic acid), 1672 cm^{-1} ($ν_{as}$C=O, salicylic acid), 1631-1443 cm^{-1} (νC=C, νC=N, NH_2 and C-H in-plane bend), 1380 cm^{-1} ($ν_{sym}$C=O, salicylic acid), 1328 (OH in-plane bend), 1239-1030 cm^{-1} (νC-N and νC-O); 759-706 cm^{-1} (=C-H out-of-plane bend), 658 cm^{-1} (in-plane ring deformation).

2. Single-Crystal (SCXRD) and Powder X-Ray Diffraction (PXRD) measurements

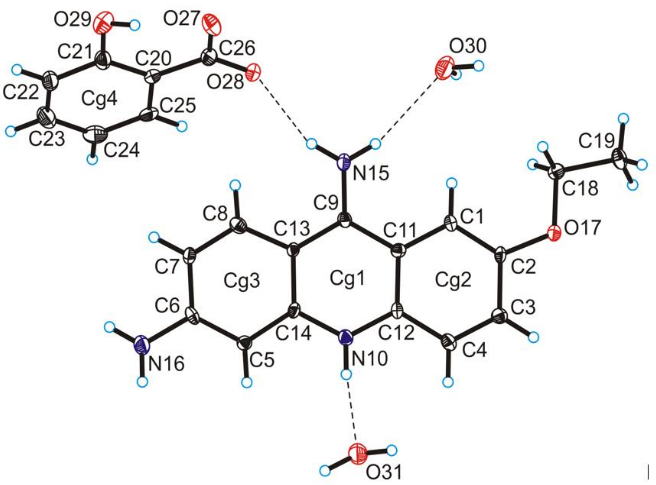

Figure 4S. Molecular structure of compound **1** showing the atom-labelling scheme (Cg1, Cg2, Cg3 and Cg4 denote the ring centroids; hydrogen bonds are represented by dashed lines).

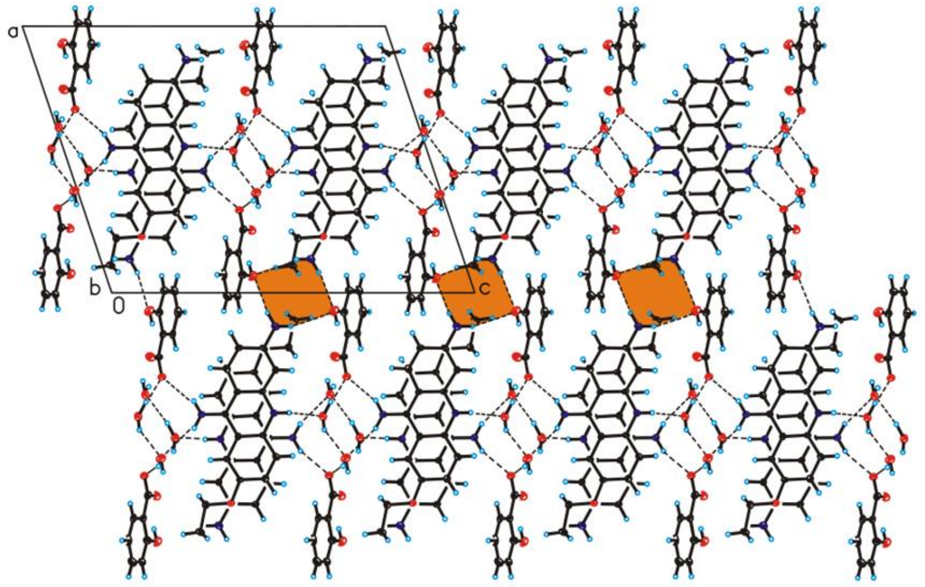

Figure 5S. Crystal packing and supramolecular synthon [···H–N–H···O···]$_2$ (highlighted in orange) in compound **1** viewed along *b*-axis (hydrogen bonds are represented by dashed lines).

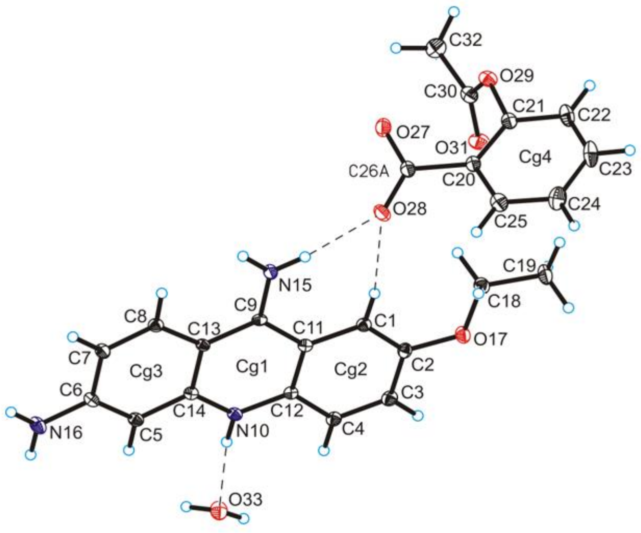

Figure 6S. Molecular structure of compound **2** showing the atom-labelling scheme (Cg1, Cg2, Cg3 and Cg4 denote the ring centroids; hydrogen bonds are represented by dashed lines).

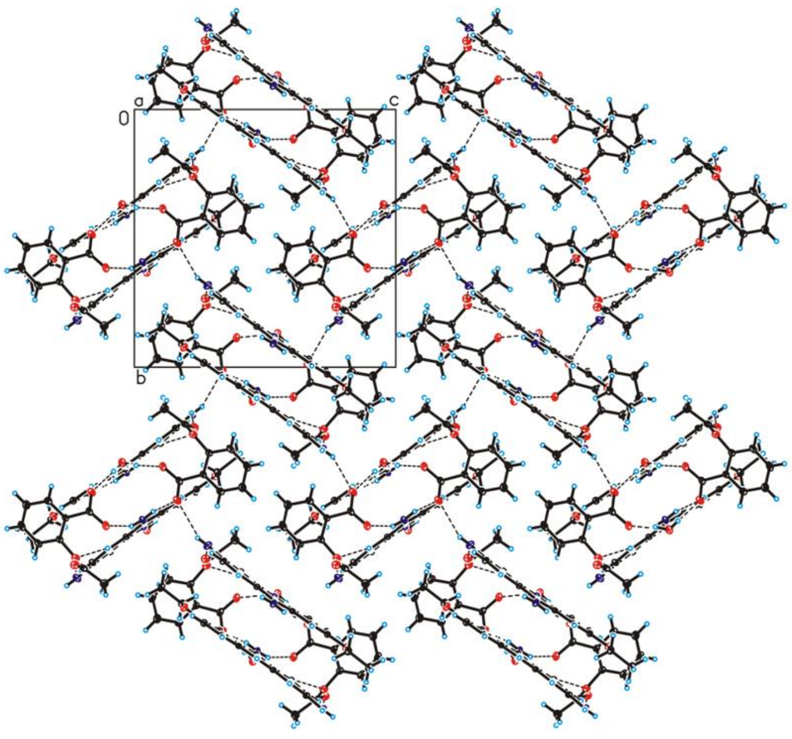

Figure 7S. Crystal packing in compound **2** viewed along *a*-axis (hydrogen bonds are represented by dashed lines).

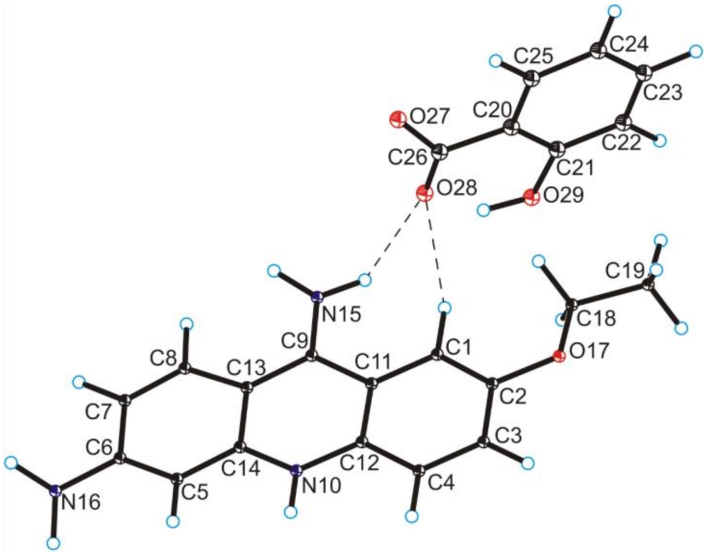

Figure 8S. Molecular structure of compound **3** showing the atom-labelling scheme (hydrogen bonds are represented by dashed lines).

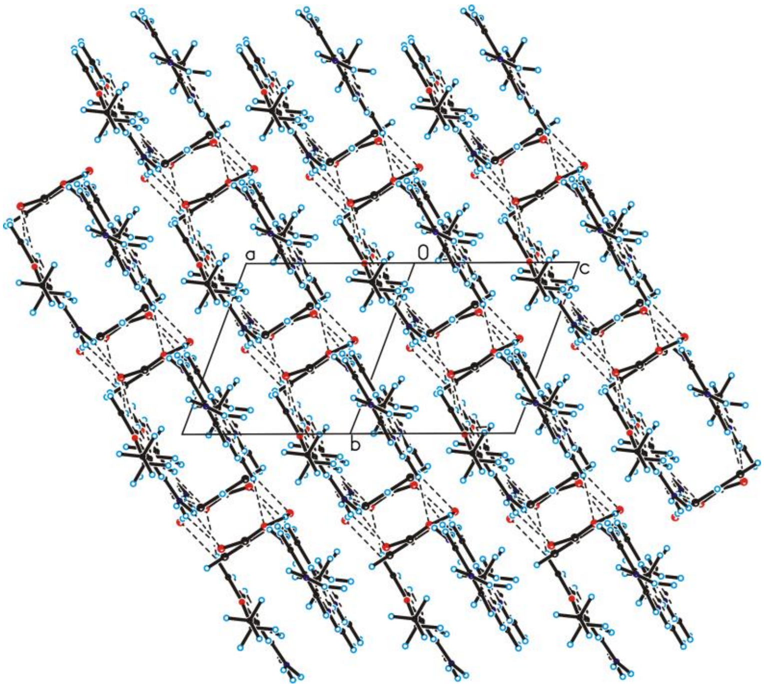

Figure 9S. Crystal packing in compound **3** viewed along [1 0 1] direction (hydrogen bonds are represented by dashed lines).

Table 1S. Crystal data and structure refinement for compounds **1**–**3**.

**Compound**	**1**	**2**	**3**
Chemical formula	$C_{22}H_{25}N_3O_6$	$C_{24}H_{25}N_3O_6$	$C_{22}H_{21}N_3O_4$
Formula weight/g·mol^{-1}	427.45	451.47	391.42
Crystal system	monoclinic	monoclinic	triclinic
Space group	*P*2$_1$/c	*P*2$_1$/n	*P-1*
a/Å	15.5334(14)	10.2917(2)	13.8453(12)
b/Å	6.8292(5)	14.6195(5)	8.6732(5)
c/Å	20.561(2)	15.0237(5)	8.6224(5)
α/°	90	90	115.695(3)
β/°	108.996(11)	93.827(3)	94.259(4)
γ/°	90	90	90.291(5)
V/Å^3	2062.3(4)	2255.44(11)	929.6(4)
Z	4	4	2
T/K	295(2)	295(2)	295(2)
λ_{Cu}/Å	1.54184	1.54184	1.5406
ρ_{calc}/g·cm^{-3}	1.377	1.330	1.398
F(000)	904	952	
μ/mm^{-1}	0.841	0.801	
θ range/°	4.549-67.998	4.224-67.269	
Reflections collected	21325	24198	
Reflections unique	3684 [R_{int} = 0.0965]	4000 [R_{int} = 0.0445]	
Data/restraints/parameters	3684/3/311	4000/1/321	
Goodness of fit on F^2	1.029	1.092	
Final R_1 value (I>2σ(I))	0.0674	0.0799	
Final wR_2 value (I>2σ(I))	0.1644	0.2431	
Final R_1 value (all data)	0.1391	0.0950	
Final wR_2 value (all data)	0.2122	0.2550	
CCDC number	2279198	2279199	2280612

Table 2S. Hydrogen bonds geometry for compounds **1**–**3**.

Compound	D–H···A	d(D–H) [Å]	d(H···A) [Å]	d(D···A) [Å]	∠D–H···A [°]
**1**	N10–H10···O31	0.96(3)	1.80(3)	2.761(4)	173(2)
	N15–H15A···O28	0.89(5)	2.08(5)	2.940(5)	163(4)
	N15–H15B···O30	0.86(3)	2.42(3)	3.255(5)	164(3)
	N16–H16A···O29^{i}	0.96(4)	2.58(4)	3.492(5)	158(4)
	N16–H16B···O29^{ii}	0.86(5)	2.50(5)	3.326(5)	161(3)
	O30–H30A···O27^{iii}	0.91(5)	2.12(5)	2.995(5)	162(5)
	O30–H30B···O28^{iv}	0.93(2)	2.38(3)	3.082(6)	132(4)
	O31–H31A···O28^{i}	0.99(8)	1.70(8)	2.690(5)	174(7)
	O31–H31B···O30^{i}	0.80(8)	2.02(8)	2.807(5)	168(6)
Symmetry code: (i) x, 1/2-y, -1/2+z; (ii) -x ,1/2+y, 1/2-z; (iii) 1-x, -y, 1-z; (iv) 1-x, 1-y, 1-z.					
**2**	N10–H10···O33	0.95(4)	1.85(4)	2.797(4)	179(4)
	N15–H15A···O27^{i}	0.84(4)	2.21(4)	3.003(4)	157(4)
	N15–H15B···O28	0.90(4)	2.07(4)	2.961(4)	168(4)
	N16–H16A···O31^{ii}	0.87(5)	2.17(5)	2.999(4)	161(4)
	N16–H16B···O28^{iii}	0.84(5)	2.48(4)	3.252(5)	154(4)
	O33–H33A···O28^{iv}	0.95(6)	1.91(6)	2.863(4)	176(5)
	O33–H33B···O27^{ii}	0.87(6)	1.82(6)	2.680(4)	174(6)
	C1–H1···O28	0.93	2.51	3.421(4)	168
	C8–H8···O29^{i}	0.93	2.47	3.163(4)	131
Symmetry code: (i) 2-x, 1-y, -z; (ii) 1-x, 1-y, -z; (iii) -1/2+x, 3/2-y, -1/2+z; (iv) -1+x, y, z.					
**3**	N15–H17···O28	1.02	2.05	2.9248(3)	143
	N15–H18···O27^{i}	1.02	2.17	3.1575(3)	162
	N10–H22···O27^{ii}	1.02	1.98	2.9481(3)	159
	C4–H7···O28^{iii}	1.09	2.37	3.1978(3)	131
	C1–H8···O28	1.09	2.51	3.5697(3)	162
	C3–H9···O29^{iii}	1.09	2.42	3.4050(3)	149
	C5–H10···O27^{ii}	1.09	2.59	3.4324(3)	133
	C8–H21···O27^{i}	1.09	2.50	3.5532(3)	162
Symmetry code: (i) -x, 1-y, 3-z; (ii) -x, -y, 2-z; (iii) x, -1+y, -1+z.					

Table 3S. π–π interactions for compounds **1**–**3**.

Compound	CgI[a]	CgJ[a]	CgI···CgJ[b] [Å]	Dihedral angle[c] [°]	Interplanar distance[d] [Å]	Offset[e] [Å]
**1**	1	1^{v}	3.6937(16)	1.83(13)	3.4257(11)	1.437
	1	2^{v}	3.5857(16)	1.38(13)	3.4166(11)	1.142
	2	1^{v}	3.6124(16)	1.38(13)	3.4294(11)	1.187
	2	3^{v}	3.6733(17)	1.65(13)	3.4274(11)	1.409
	2	3^{vi}	3.7389(17)	1.65(13)	3.3976(11)	1.474
Symmetry code: (v) 1-x, -1/2+y, 1/2-z; (vi) 1-x, 1/2+y, 1/2-z.						
**2**	1	1^{ii}	3.4812(17)	0.00(14)	3.3250(12)	1.031
	1	2^{ii}	3.6695(17)	0.82(14)	3.3395(12)	1.481
	2	3^{ii}	3.5909(19)	3.44(15)	3.3743(13)	1.032
Symmetry code: (ii) 1-x, 1-y, -z.						
**3**	1	1^{ii}	3.7995(3)	0	3.4216	1.652
	1	2^{ii}	3.6768(3)	1	3.4327	1.294
	2	3^{ii}	3.8773(3)	3	3.4507	1.661
Symmetry code: (ii) -x, -y, 2-z.						

[a] Cg represents the centre of gravity of the rings. [b] Cg···Cg is the distance between ring centroids. [c] The dihedral angle is that between the mean planes of CgI and CgJ. [d] The interplanar distance is the perpendicular distance from CgI to ring J. [e] The offset is the perpendicular distance from ring I to ring J.

Table 4S. C–H···π interactions geometry for compounds **1–3**.

Compound	C–H···Cg	d(H···Cg) [Å]	d(C···Cg) [Å]	∠C–H···Cg [°]
**1**	C19–H19A···Cg4^{iv}	2.88	3.750(4)	151
Symmetry code: (iv) 1-x, 1-y, 1-z.				
**2**	C3–H3···Cg4^{iv}	2.81	3.670(4)	153
Symmetry code: (iv) -1+x, y, z.				
**3**	C25–H2···Cg2^{iv}	2.75	3.6584(3)	140
Symmetry code: (iv) x, y, 1+z.				

3. MALDI-TOF mass spectra

Positive-ion mode MALDI-TOF mass spectra were obtained using a Bruker Biflex III spectrometer with 2,5-dihydroxybenzoic acid matric.

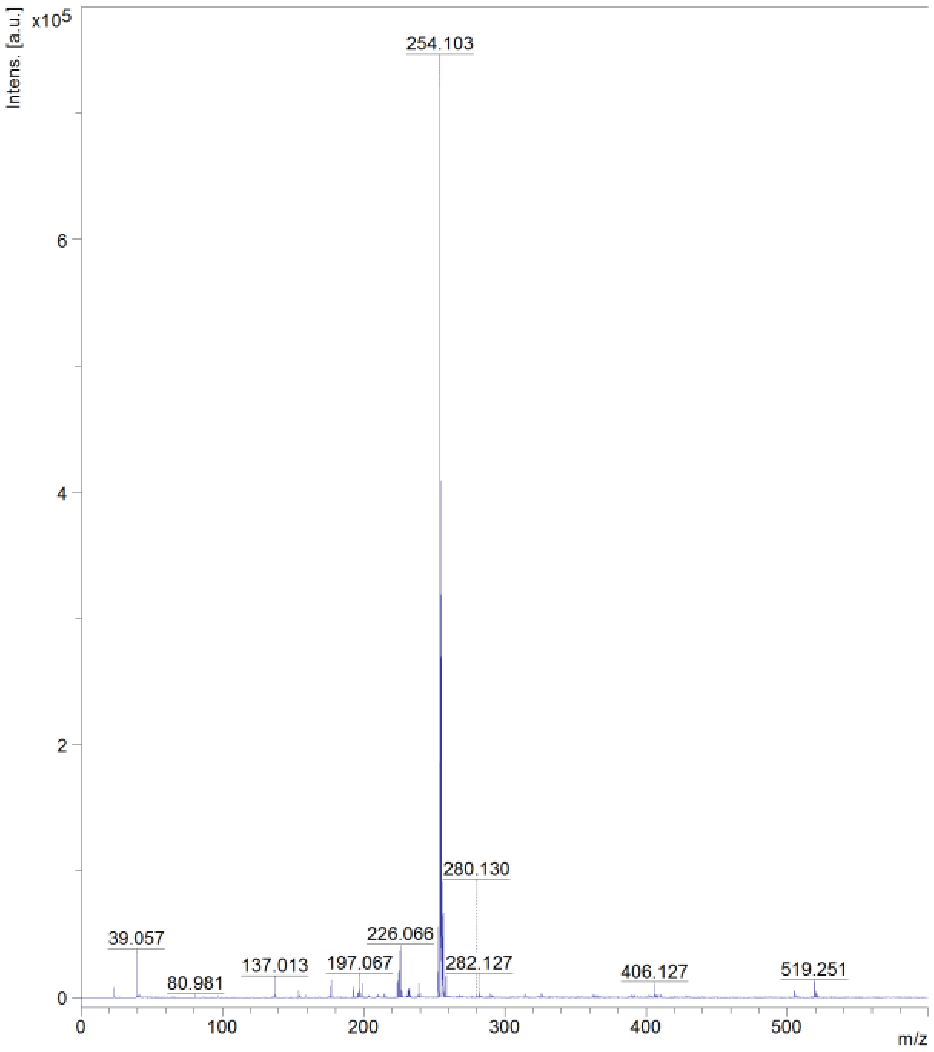

Figure 10S. MALDI-TOF mass spectra for compound **1** (calculated for $[C_{15}H_{16}N_3O]^+$: 254.312; found: 254.103).

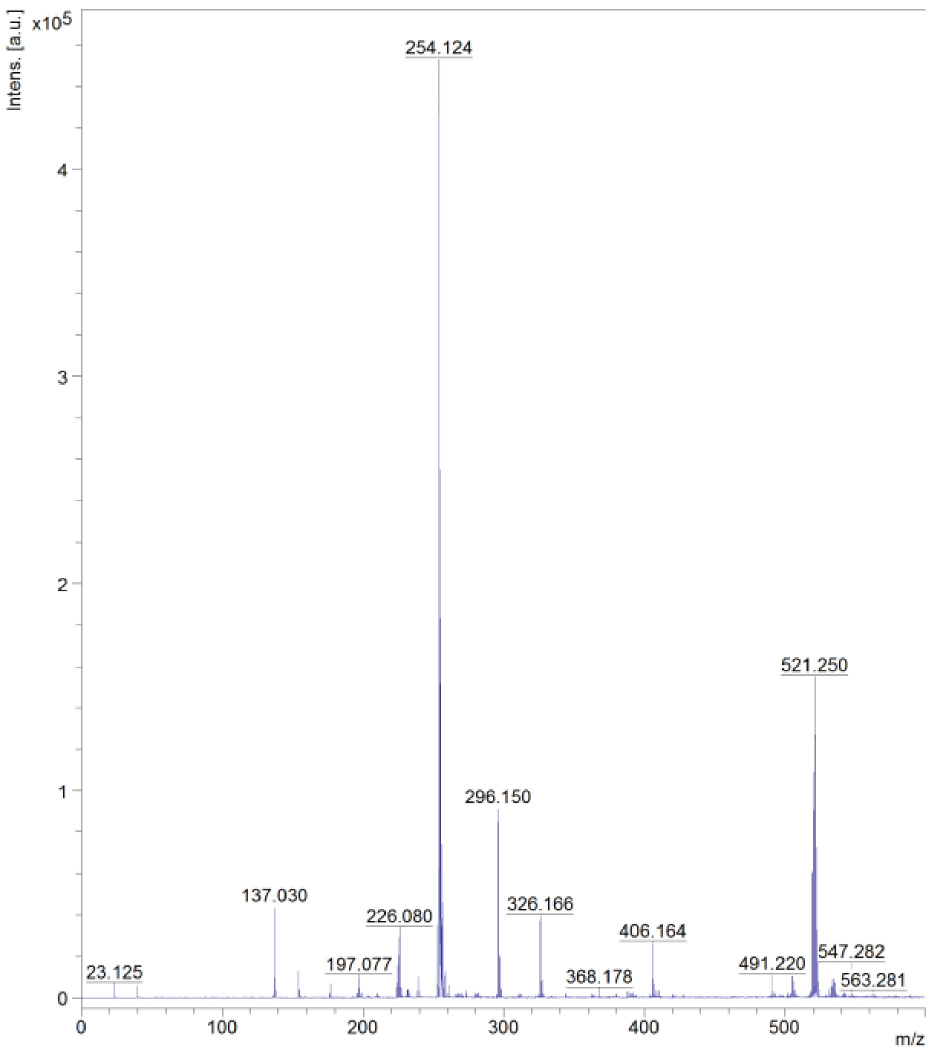

Figure 11S. MALDI-TOF mass spectra for compound **2** (calculated for $[C_{15}H_{16}N_3O]^+$: 254.312; found: 254.124).

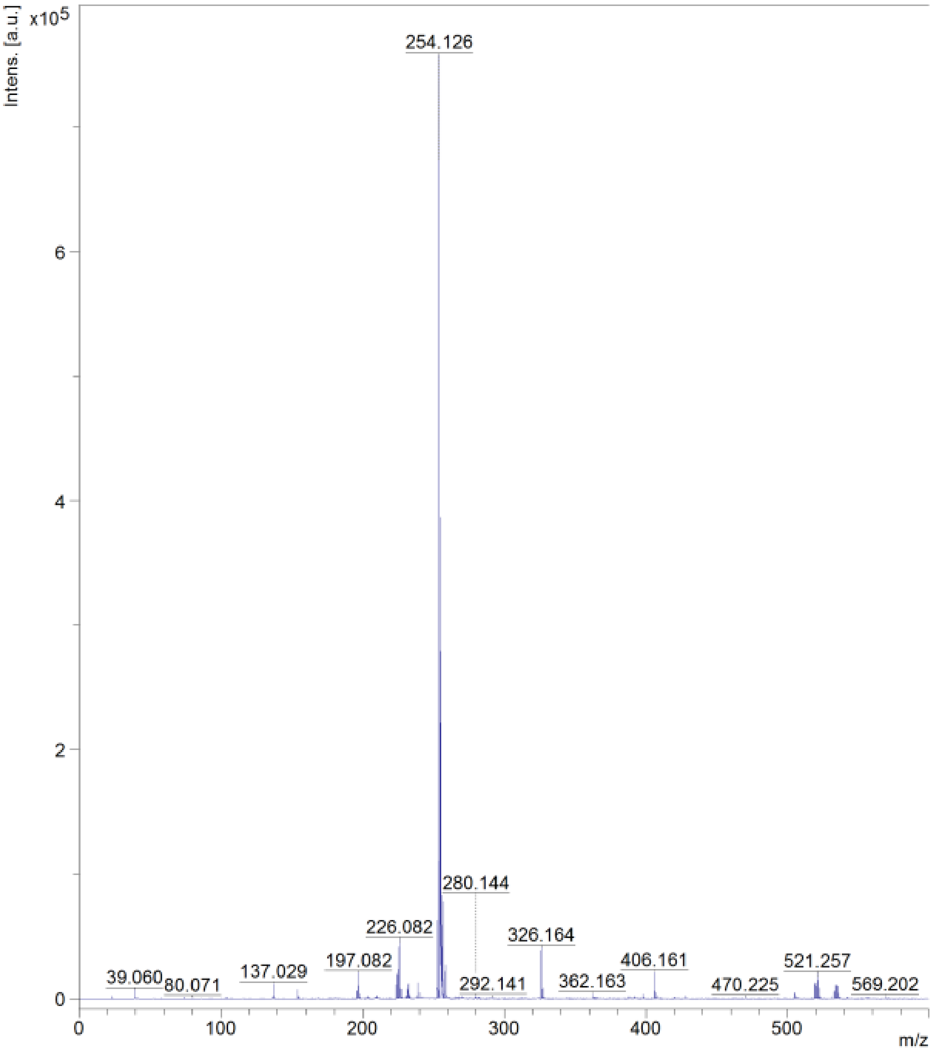

Figure 12S. MALDI-TOF mass spectra for compound **3** (calculated for $[C_{15}H_{16}N_3O]^+$: 254.312; found: 254.126).

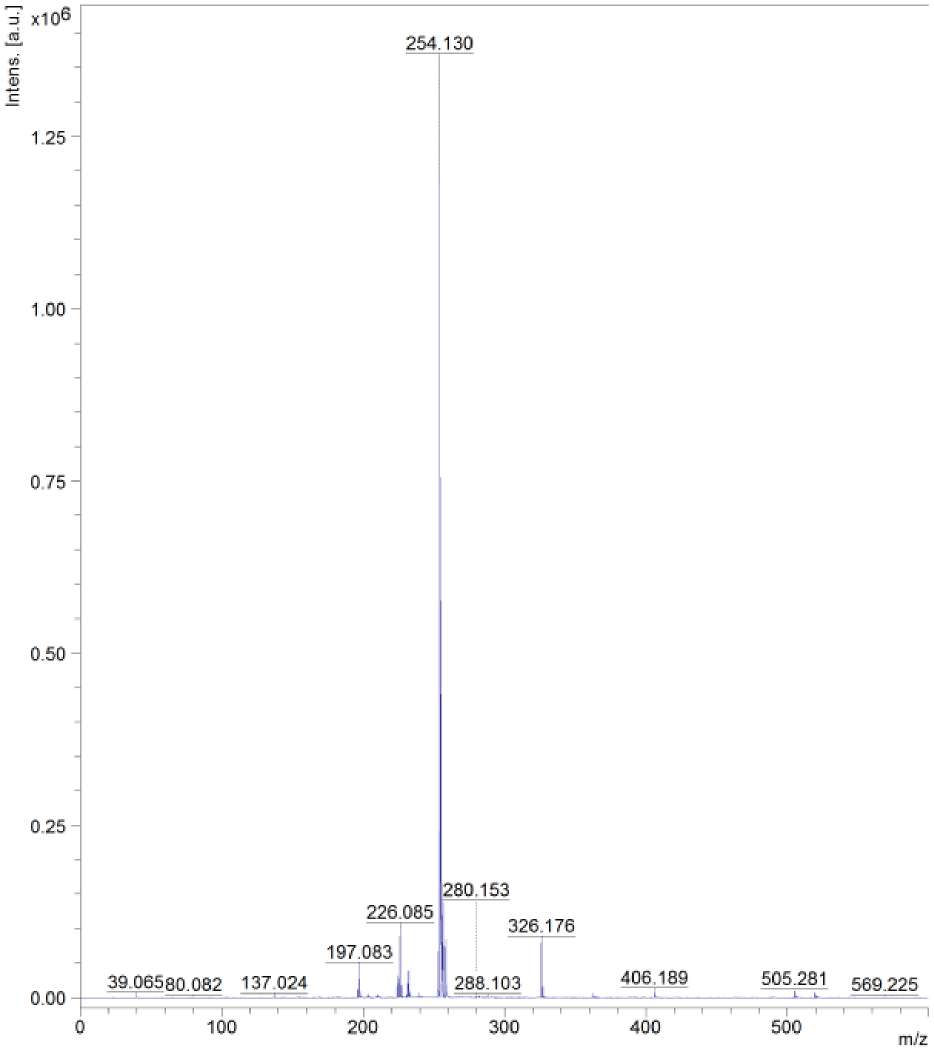

Figure 13S. MALDI-TOF mass spectra for mechanical mixture of ethacridine and salicylic acid (found: 254.130).

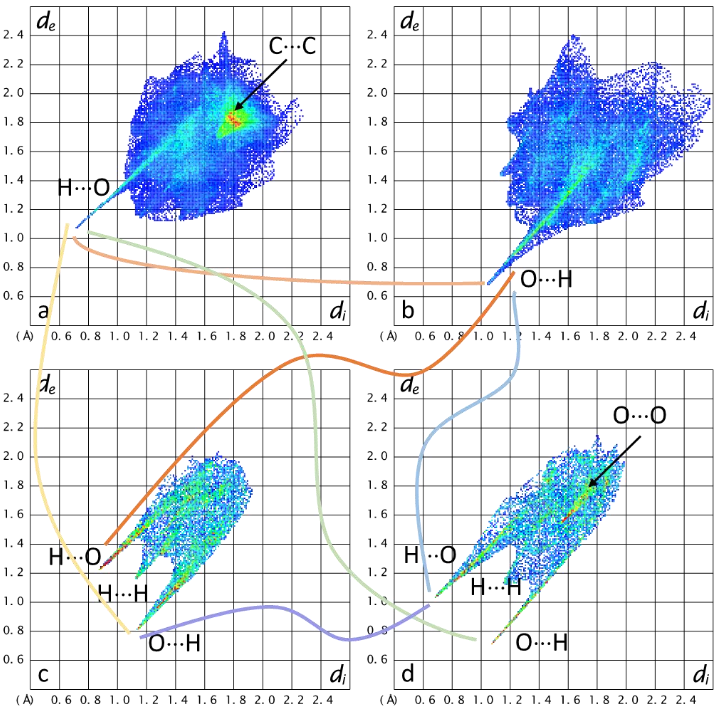

Figure 14S. Fingerprint plots for compound **1.** a) 6,9-diamino-2-ethoxyacridinium b) 2-hydroxybenzoate c) water #1 d) water #2.

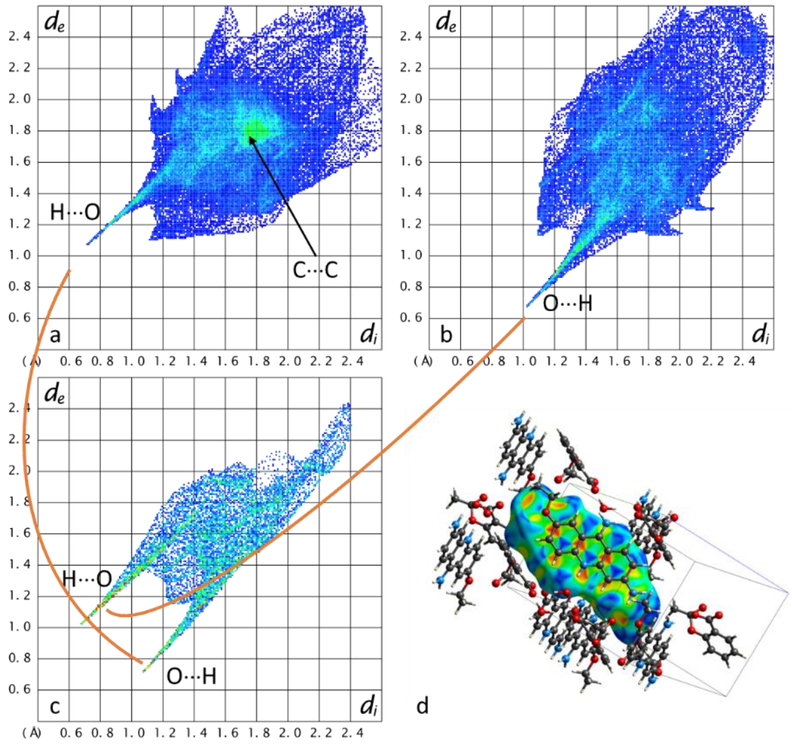

Figure 15S. Fingerprint plots for compound **2.** a) 6,9-diamino-2-ethoxyacridinium b) 2-hydroxybenzoic acid c) water. d) Shape index plotted on the Hirshfeld surface of 6,9-diamino-2-ethoxyacridinium.

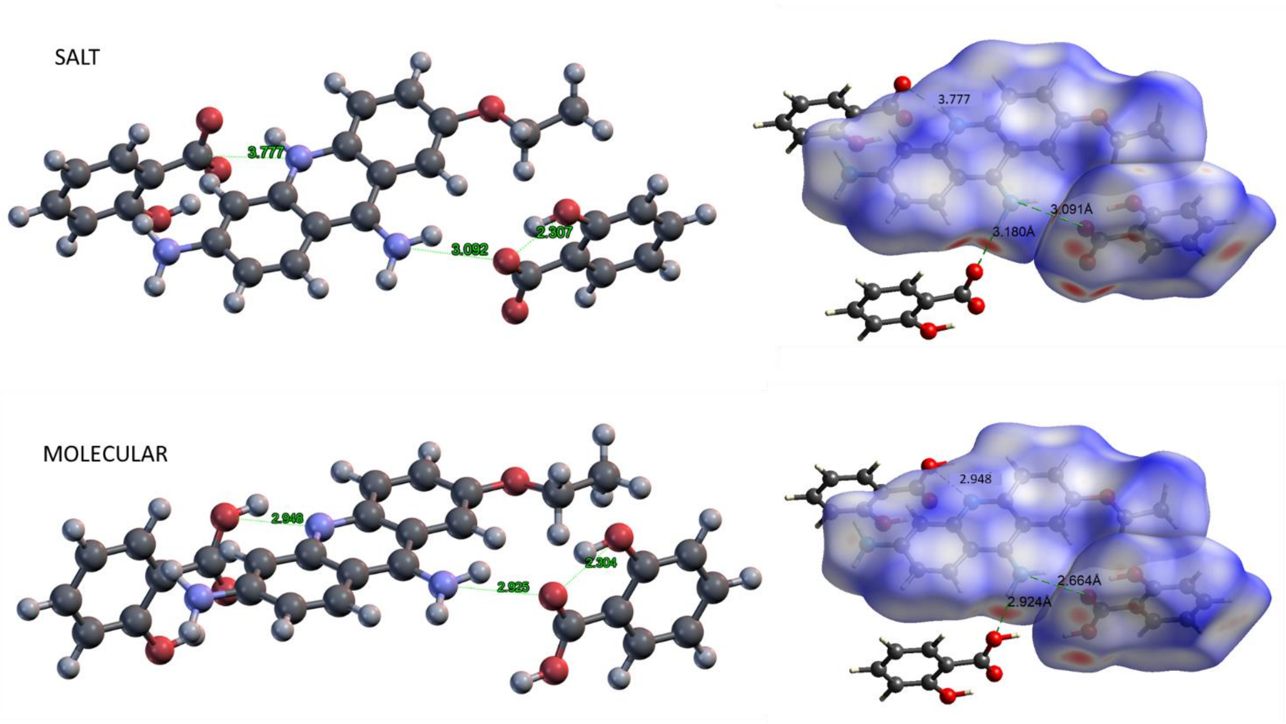

Figure 16S. Molecular structure (left) with relevant interatomic distances and Hirshfeld surfaces (right) for compound **3** in salt form (top) and molecular form (bottom).
